# New data on Amynodontidae (Mammalia, Perissodactyla) from Eastern Europe: Phylogenetic and palaeobiogeographic implications around the Eocene-Oligocene transition

**DOI:** 10.1371/journal.pone.0193774

**Published:** 2018-04-18

**Authors:** Jérémy Tissier, Damien Becker, Vlad Codrea, Loïc Costeur, Cristina Fărcaş, Alexandru Solomon, Marton Venczel, Olivier Maridet

**Affiliations:** 1 Jurassica Museum, Porrentruy, Switzerland; 2 Department of Geosciences, University of Fribourg, Fribourg, Switzerland; 3 Department of Geology, Faculty of Biology-Geology, Babeş-Bolyai University, Cluj-Napoca, Romania; 4 Naturhistorisches Museum Basel, Basel, Switzerland; 5 Faculty of Environment Science, Babeş-Bolyai University, Cluj-Napoca, Romania; 6 Department of Natural History, Ţării Crişurilor Museum, Oradea, Romania; Royal Belgian Institute of Natural Sciences, BELGIUM

## Abstract

Amynodontidae is a family of Rhinocerotoidea (Mammalia, Perissodactyla) known from the late Early Eocene to the latest Oligocene, in North America and Eurasia. European Amynodontidae are very rare, and all remains belong almost exclusively to a single post—Grande Coupure genus from the Oligocene, *Cadurcotherium*. The “Grande Coupure” defines an extinctions and dispersal-generated originations event in Europe that is nearly contemporaneous with the Eocene-Oligocene transition. Perissodactyls are one of the major groups affected by this event: Palaeotheriidae went almost extinct during this crisis, whereas Rhinocerotidae appeared for the first time in Europe. Study of fossiliferous Eastern-European localities from this age is crucial for the understanding of this crisis. We report here three new localities of Amynodontidae in Eastern Europe. Two of them are dated from the Eocene (Morlaca, Romania; Dorog, Hungary), whereas the other is either Late Eocene or Early Oligocene (Dobârca, Romania). The skull from this latter locality belongs unexpectedly to the same individual as a previously described mandible attributed to *“Cadurcodon” zimborensis*. As a result, this specimen can be allocated to its proper locality, Dobârca, and is assigned to a new genus, *Sellamynodon* gen. nov. It is characterised by an extraordinary growth of the nuchal crest, a unique character among amynodontids. Along with this remarkable material from Dobârca, two specimens from another Romanian locality, Morlaca, have been recently discovered and are dated from the Late Eocene. They belong, as well as new material from Dorog (Middle Eocene, Hungary), to the genus *Amynodontopsis*, also found in North America. The new Hungarian material represents the earliest occurrence of Amynodontidae in Europe. New phylogenetic hypotheses of Rhinocerotoidea are proposed, including the new material presented here, and show that Amynodontidae may be closer to the polyphyletic family ʽHyracodontidaeʼ than to Rhinocerotidae. Amynodontidae, with their deep preorbital fossa and extremely reduced premolars, display in fact a very derived condition, compared to rhinocerotids.

## Introduction

Amynodontidae is an extinct family of Perissodactyla that is generally included in the Rhinocerotoidea (e.g. [1–4]). They were most diverse during the Eocene, in Asia and North America [2,4–6]. The oldest representatives of the family Amynodontidae are from the Middle Eocene of China [7,8] while the most recent are from the latest Oligocene of Europe and Pakistan (and not Early Miocene as previously suggested; [9,10]). Only two species have been reported in the Eocene of Eastern Europe: *Cadurcodon ardynensis* in the Late Eocene of Bulgaria [11], and *Amynodon hungaricus* in Hungary, for which a Late Eocene age is assumed [12]. Yet, only the genus *Cadurcotherium* is recorded in Western Europe, during the Early Oligocene [13–16]. This occurrence is related to a major extinction-origination event called the “Grande Coupure” [17], which roughly happens during the Eocene-Oligocene transition (34 Ma; [18]).

The phylogenetic position of Amynodontidae within perissodactyls is quite unstable [19]. The potential relationships of this family, outside the rhinocerotoids, have only been phylogenetically tested by including two amynodontid taxa (*Rostriamynodon grangeri*, and the type genus of the family: *Amynodon*) [20–23]. However, the monophyly of Amynodontidae and the synapomorphies of this clade within Rhinocerotoidea are well-established [2]: quadratic M3, loss of upper and lower P1, enlarged canines, elongated talonids and presence of a preorbital fossa. A revision of amynodontids has recently been published [4], in which a new *Cadurcodon* species from the Late or middle-Late Eocene of China is described. They proposed the most complete phylogeny of this group to date (16 amynodontid taxa) and recognized two derived clades: the Metamynodontini (including *Paramynodon*, *Megalamynodon* and *Metamynodon*) and the Cadurcodontini (including *Procadurcodon*, *Zaisanamynodon*, *Cadurcodon* and *Cadurcotherium*).

**Fig 1:**
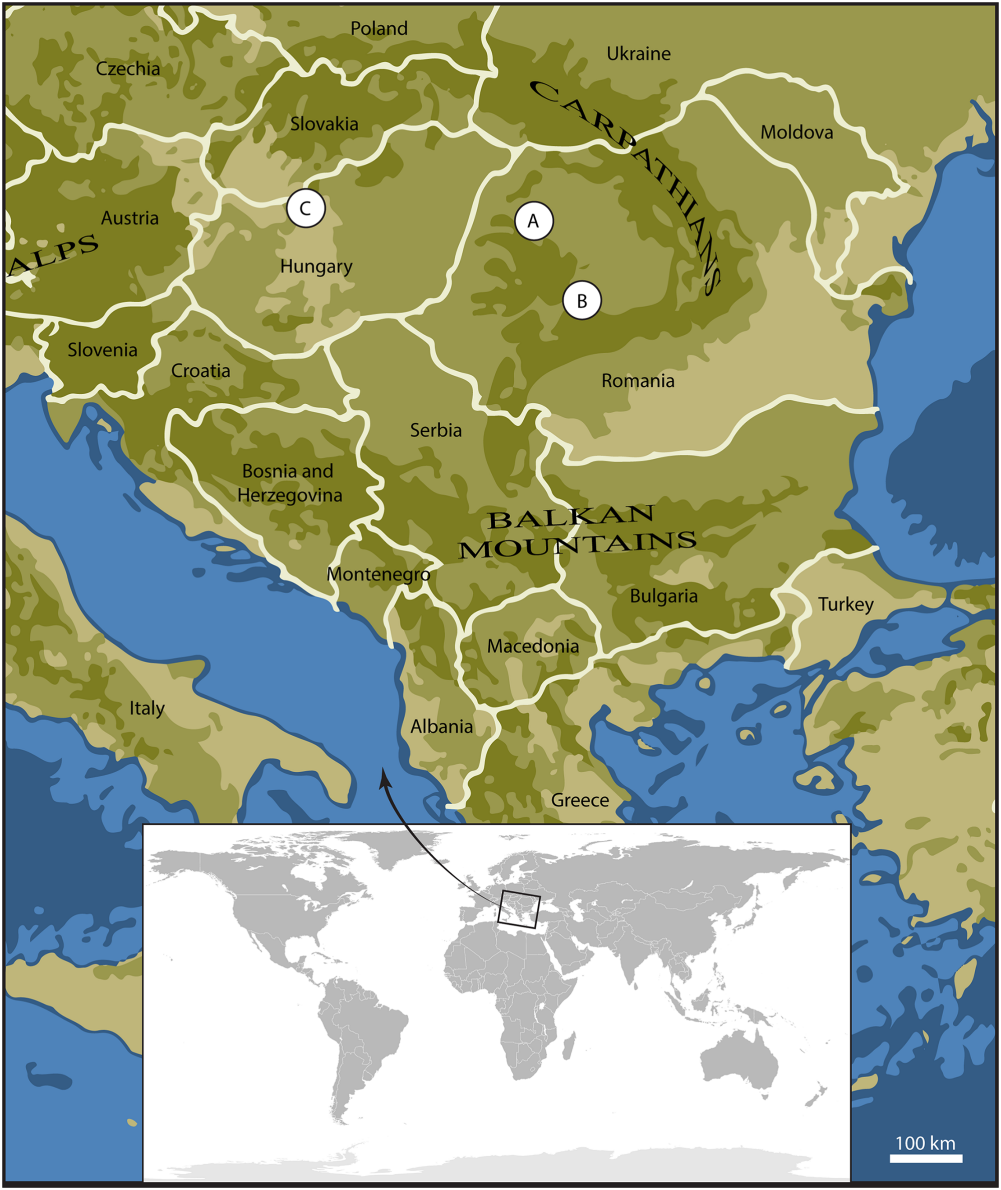
Geographical setting of the new amynodontid localities in Eastern Europe. A: Morlaca (Romania), Priabonian. B: Dobârca (Romania), Priabonian-Rupelian. C: Dorog (Hungary), late Lutetian-Bartonian.

We report here new material of Amynodontidae from three localities in South-Eastern Europe (Fig 1): Dorog (Middle Eocene, Hungary), Morlaca (latest Eocene, Romania) and Dobârca (Late Eocene or Early Oligocene, Romania). These specimens are included in a large scale cladistic analysis and results are discussed in the palaeobiogeographical context of the Grande Coupure in Europe.

## Material and methods

### Material

The new material from Hungary (HNHM PAL 2017.54.1, a maxillary fragment with M2-3) is stored in the Hungarian Natural History Museum (Budapest, Hungary). The Romanian material is stored in the Paleontology-Stratigraphy Museum of the Babes-Bolyai University (Cluj-Napoca, Romania). It encompasses a right maxilla fragment with complete molar row (UBB MPS V545) and a small mandibular fragment with m1/2 (UBB MPS V546) from Morlaca, as well as a fragmentary skull associated to its mandible (UBB MPS 15795) from Dobârca.

The specimens from Dobârca and Morlaca have been surface-scanned with a structured-light scanner (Artec Space Spider) and reconstructed with Artec Studio 10 Professional. The 3D models of these specimens are available at MorphoMuseuM.com [24].

### Geology

The specimen from Hungary was given to Miklós Kretzoi during his lifetime, though he never mentioned or described it. It is labelled “Dorog substratum” and therefore we assume that it comes from the Dorog coal Formation in Dorog Basin (Hungary). This is confirmed by the especially dark and carbonaceous preservation of the specimen. This formation is either latest Lutetian or Bartonian in age (late Middle Eocene; [25,26]).

The Morlaca locality (Cluj district, Romania) belongs to the Valea Nadăşului Formation [27], dated from the Priabonian [28,29].

The mandible from Dobârca was originally described as coming from the Zimbor Strata (Sălaj County, Romania) and assigned to *“Cadurcodon” zimborensis* [30], but its allocation was corrected as coming from the Dobârca locality (Sibiu County) [31]. Indeed, the recent discovery of the matrix counter-impression of the mandible in the collections of the UBB revealed that it was labelled “Dobârca”. Furthermore, it permitted the association with a previously unpublished skull, which was properly labelled “Dobârca”, and articulates perfectly with the mandible. On the basis of these new data, we assume that both belong to the same individual and thus come from that same locality. However, its age remains uncertain. Dobârca is located at the boundary between the Transylvanian Basin and Southern Carpathians. In Dobârca Valley, fluvial red beds are in contact with the metamorphic rocks of the Cândrelului Mountains and overlaid by grey-bluish sandstone interbeddings [32]. The amynodontid remains were probably unearthed from this layer, but no other fossils are recorded in it. The succession can be compared with the red clay recorded eastward in Rodului Valley, at Apoldu de Sus. There, Mészáros et al. [33], mentioned a pile of “red clay, gray-greenish clayish and mica sandstone” with *Chara*, considered by these geologists as “Burdigalian-Helvetian” (i.e. Lower Miocene), laying on Upper Eocene (Priabonian) deposits. On the southern border of the Transylvanian Basin, Eocene lithostratigraphic units were coined by Mészáros [34] at Turnu Rosu-Porcești, near Sibiu. The youngest one, named Valea Nișului Formation is Priabonian-Lower Oligocene. The Oligocene rocks refer to “sandstone with fish scales”, bearing also plant remains. Visibly the rock succession of Dobârca is not identical neither with the one of Turnu Roșu-Porcesti, nor with the Ighiu Formation (Late Eocene-Early Oligocene; [35]) from the southwestern area of the basin. Considering the rock succession from Apoldu de Sus as the most convenient for a comparison with the one of Dobârca, one may consider the sandstone bearing the amynodont remains as late Eocene or Early Oligocene in age, which can be included for instance in Valea Nișului Formation. The similar rocks of Apoldu are herein considered as Oligocene, not Miocene [32,33].

### Nomenclatural acts

The electronic edition of this article conforms to the requirements of the amended International Code of Zoological Nomenclature, and hence the new names contained herein are available under that Code from the electronic edition of this article. This published work and the nomenclatural acts it contains have been registered in ZooBank, the online registration system for the ICZN. The ZooBank LSIDs (Life Science Identifiers) can be resolved and the associated information viewed through any standard web browser by appending the LSID to the prefix “http://zoobank.org/”. The LSID for this publication is: urn:lsid:zoobank.org:pub:6BBF9472-2A60-4C51-BD82-3156FD9A4827. The electronic edition of this work was published in a journal with an ISSN, and has been archived and is available from the following digital repositories: PubMed Central, LOCKSS.

### Anatomical terminology and characters

Cranial and dental terminology is from Antoine [36]. Anatomical features described follow the same sequence as Antoine [36,37], completed by characters newly observed in this work. Capital letters are used for upper teeth (I, C, P, M), and lower-case letters for lower teeth (i, c, p, m). Italicized numbers between parentheses refer to the character number in the data matrix (see S1 File). Dental and cranial measurements are taken according to Uhlig [38] and Antoine [36], respectively. Dimensions are given in mm.

### Institutional abbreviations

HNHM “Hungarian Natural History Museum” (Budapest, Hungary), IVPP “Institute of Vertebrate Palaeontology and Palaeoanthropology” (Beijing, China), MHNT “Muséum de Toulouse” (Toulouse, France), MJSN “JURASSICA Museum” (formerly “Musée jurassien des sciences naturelles”; Porrentruy, Switzerland), NMB “Naturhistorisches Museum Basel” (Basel, Switzerland), SMNS “Staatliches Museum für Naturkunde Stuttgart” (Stuttgart, Germany), UBB “Universitatea Babeș-Bolyai” (Cluj-Napoca, Romania).

### Phylogenetic analysis

The morphological characters matrix is based on a published matrix [36,37], and is available in Supporting information (S1 File) in NEXUS and TNT format. Fifteen characters were added to better resolve the relationships within Amynodontidae (for which the original data matrix was not created specifically). Eight of them (*290–297*) are taken from another matrix [4]: characters 8, 9, 16, 23, 25, 39, 41 and 45, and seven are completely new:

- (*283*): C = 0, reduced; 1, developed; 2, strong
- (*284*): c = 0, reduced; 1, developed; 2, strong
- (*285*): preorbital fossa = 0, absent or reduced; 1, present
- (*286*): mandible: space between condylar and coronoid processes = 0, short (V-shaped); 1, wide (U-shaped)
- (*287*): mandible: condylar process = 0, high; 1, low
- (*288*): m3 talonid = 0, equal or smaller than trigonid; 1, longer than trigonid
- (*289*): M3 paracone fold = 0, absent; 1, weak; 2, strong.

Characters were scored based on specimen observations and publications, including descriptions, pictures and illustrations (Table 1). The cladistic analysis was performed with the heuristic search in PAUP* version 4.0b10 and 4.0a159 [39] and with the traditional search in TNT version 1.1 [40]. In PAUP, 1000 replicates of random-addition-sequence were performed, with a TBR branch-swapping algorithm and MulTrees option in effect. Starting trees were obtained by stepwise addition and 100 trees were held at each replicate. In TNT, starting trees were obtained from Wagner trees. 1000 replicates of addition-sequence were performed using TBR algorithm and saving 100 trees per replication. Seven characters do not form a morphocline and were considered as unordered: characters (*72*), (*94*), (*102*), (*187*), (*190*), (*292*) and (*293*); all the others were ordered. Four characters have been modified from the original matrix [36,37] to form morphoclines: (*36*), (*60*), (*103*) and (*140*) (see data matrix in S1 File). Uninformative and constant characters were deleted prior to the analyses (“exclude uninf” and “exclude constant” commands in PAUP and “xinact” command in TNT); thus, 184 characters were kept during the analysis for 38 taxa. Decay index (Bremer support) was calculated with the use of TreeRot.v3 software [41] and PAUP* version 4.0b10 (TreeRot script was not compatible with PAUP* version 4.0a159).

**Table 1:** List of taxa included in the phylogenetic analysis and their coding sources.

Terminal	Character coding (source)	
	Direct observation	References
β *Aceratherium incisivum* Kaup 1832	MJSN, NMB	Antoine et al. 2010 [37]
β *Allacerops turgaica* (Borissiak 1918)		Borissiak 1918 [42]; Reshetov et al. 1993 [43]
*Amynodon advenus* (Marsh 1875)		Scott & Osborn 1890 [44]; Troxell 1921 [45]; Wilson & Schiebout 1981 [6]; Wall 1982 [46]; Campisano et al. 2014 [47]
*« Amynodon » reedi* Stock 1939		Stock 1939 [48]
*Amynodontopsis bodei* Stock 1933		Stock 1933, 1936, 1939 [48–50]; Wilson & Schiebout 1981 [6]
*Amynodontopsis* aff. *bodei*	UBB, HNHM	
*Cadurcodon ardynensis* (Osborn 1923)		Osborn 1924 [51]; Gromova 1954, 1958 [52,53]; Lucas & Emry 1996 [54]
*Cadurcodon bahoensis* Xu 1965		Xu 1965 [55]
*Cadurcodon kazakademius* Birjukov 1961		Birjukov 1961 [56]; Lucas & Emry 1996 [54]
*Cadurcodon maomingensis* Averianov et al. 2016		Averianov et al. 2016 [4]
*Cadurcotherium cayluxi* Gervais 1873	MHNT, NMB	Gervais 1873 [13]; Roman & Joleaud 1909 [14]; de Bonis 1995 [15]
*Cadurcotherium minus* Filhol 1880	MHNT, NMB, NMBE	Roman & Joleaud 1909 [14]
β *Eggysodon osborni* (Schlosser 1902)	SMNS, NMB	Schlosser 1902 [57]; Roman 1911 [58]; de Bonis & Brunet 1995 [59]; Uhlig 1999 [38]; Pandolfi 2015 [60]
β *Hyrachyus eximius* Leidy 1871		Antoine et al. 2010 [37]
β *Hyrachyus princeps* Marsh 1872		Troxell 1922 [61]; Wood 1934 [62]
β *Hyracodon nebraskensis* Leidy 1850	HNHM, MHNT	Scott 1941 [63]; Radinsky 1967 [64]
*Megalamynodon regalis* Scott 1945		Scott 1945 [65]
*Metamynodon planifrons* Scott & Osborn 1887		Scott & Osborn 1887 [66]; Troxell 1921 [67]; Scott 1941 [63]; Manning et al. 1985 [68]
β *Pappaceras confluens* Wood 1963		Wood 1963 [69]
β *« Pappaceras » meiomenus* Wang et al. 2016	IVPP	Wang et al. 2016 [23]
β *Paraceratherium bugtiense* (Pilgrim 1908)		Forster-Cooper 1911, 1913, 1923, 1924, 1934 [70–74]; Lucas & Sobus 1989 [75]
β *Paraceratherium transouralicum* (Pavlova 1922)	HNHM	Osborn 1923 [76]; Borissiak 1923 [77]; Granger & Gregory 1936 [78]; Lucas & Sobus 1989 [75]
*Paramynodon birmanicus* (Pilgrim & Cotter 1916)		Colbert 1938 [79]
β *Proeggysodon qiui* Bai & Wang 2012		Bai & Wang 2012 [80]
*Protapirus simplex* Wortman & Earle 1893		Scott 1941 [63]
β *Ronzotherium filholi* (Osborn 1900)	NMB	Antoine et al. 2010 [37]
*Rostriamynodon grangeri* Wall & Manning 1986		Wall & Manning 1986 [19]
*Sellamynodon zimborensis* (Codrea & Şuraru 1989)	UBB	Codrea & Şuraru 1989 [30]
*Sharamynodon mongoliensis* (Osborn 1936)	IVPP	Osborn 1936 [81]; Young 1937 [82]; Lucas & Emry 2001 [83]
*Sianodon gaowangouensis* Li 2003		Li 2003 [8]
*Tapirus terrestris* (von Linnaeus 1758)	NMB	Antoine et al. 2010 [37]
β *Teletaceras radinskyi* Hanson 1989		Hanson 1989 [84]
β *Trigonias osborni* Lucas 1900		Antoine et al. 2010 [37]
β *Triplopus obliquidens* (Scott & Osborn 1887)		Scott & Osborn 1890 [44]; Peterson 1919 [85]; Radinsky 1967 [64]
β *Uintaceras radinskyi* Holbrook & Lucas 1997		Holbrook & Lucas 1997 [86]
β *Urtinotherium intermedium* (Chiu 1962)		Chiu 1962 [87]; Qiu & Wang 2007 [88]
*Zaisanamynodon borisovi* Belyaeva 1971		Lucas et al. 1996 [89]; Lucas 2006 [3]
*Zaisanamynodon protheroi* Lucas 2006		Lucas 2006 [3]

β symbol before taxon name indicates that it belongs to the “branching-group”.

The taxonomic sampling includes five taxa previously coded [37]: *Tapirus terrestris* (considered as outgroup for this analysis), *Hyrachyus eximius* and three unambiguous Rhinocerotidae (considered part of the branching-group [36, 37]): *Trigonias osborni*, *Ronzotherium filholi* and *Aceratherium incisivum*. Thirty-three new terminals were added to the matrix, including the new amynodontid remains (two taxa), seventeen amymodontids, one tapiroid (*Protapirus simplex* [= *Protapirus validus*]; also considered as outgroup), and the branching-group. Terminals in the branching-group have been chosen to test the monophyly of Amynodontidae as well as their position inside the Rhinocerotoidea. Therefore, they have to represent all major groups of rhinocerotodoids (Rhinocerotidae, Eggysodontidae etc.). It includes another species of *Hyrachyus* (*H*. *princeps*; though it was considered a junior synonym of *H*. *eximius* [90], we consider it as valid based on several anatomical differences), two equivocal early diverging rhinocerotids (*Uintaceras radinskyi* and *Teletaceras radinskyi*) and 10 hypothetical “hyracodontid” species. However, the goal of this analysis is not to resolve the phylogeny of Rhinocerotoidea, which is beyond the scope of this study. The branching-group should only lead to a better polarization of characters, which is necessary here because the two outgroups are both tapiroids and thus act as a single monophyletic outgroup. Terminals in the ingroup (i.e. our group of interest) have been chosen to represent nearly all amynodontids, to allow the placement of the new material. The taxonomic sampling and coding sources are provided in Table 1.

### Remarks on taxonomy

The taxonomy of Amynodontidae was recently revised [4], and we follow these suggestions on most of the taxonomic considerations. Therefore, we consider *Lushiamynodon* Chow & Xu, 1965 a synonym of *Sharamynodon* Kretzoi, 1942 and *Sianodon* Xu, 1965 (with type species *S*. *bahoensis*) as a junior synonym of *Cadurcodon* Kretzoi, 1942. We also agree not to recognize *“Sianodon” ulausuensis* and *“Sianodon” gaowangouensis* as belonging to *Cadurcodon*, since both have three upper incisors, whereas *Cadurcodon* only has two. *“Sianodon” ulausuensis* Xu, 1966 has not been included in the analysis because it acted as a “wildcard” taxon, probably because the referred cranial specimen is much distorted and most teeth are not preserved or incomplete. *“Sianodon” gaowangouensis* however has been included, which was represented by a palate specimen bearing complete dental series [8]. However, we follow Lucas (2006; [3]) and consider *Zaisanamynodon protheroi* as valid but *Procadurcodon orientalis* as a *nomen dubium* (*nomen vanum*).

*Metamynodon bugtiensis* Forster-Cooper, 1922, from the Bugti deposits in Baluchistan, was described as a gigantic amynodontid [91], based on the belief that the last erupting tooth (still far in alveoli) was a M3, mostly because there was no space behind it for another tooth. Yet, dissecting the specimen more than 10 years later (Figs 20A and 22 in [73]), it was revealed that what was considered as P4 and M1 were in fact DP3 and DP4. The first preserved tooth is then a DP1, and not a P2 or DP2. This is also congruent with the fact that the M1 is usually the most worn tooth (e.g. Fig 19.5 in [75]). This specimen should therefore be referred to an aberrant form of *Paraceratherium bugtiense* lacking the M3 [73,92].

## Results

### Systematic palaeontology

Mammalia Linnaeus, 1758 [93]

Perissodactyla Owen, 1848 [94]

Rhinocerotoidea Gray, 1821 [95]

Amynodontidae Scott & Osborn, 1883 [96]

Metamynodontini Kretzoi, 1942 [92]

*Sellamynodon* gen. nov. urn:lsid:zoobank.org:act:8925D805-AD55-4FD9-8663-48AA691250B9

1989 *“Cadurcodon”*; Codrea & Şuraru [30]: 319–338

### Etymology

Contraction of Latin word *sella*, saddle, in reference to the saddle-shaped dorsal profile of the skull and *amynodon*, a frequent suffix in the genus names of Amynodontidae.

### Type species

*“Cadurcodon” zimborensis* Codrea & Şuraru, 1989 [30]

### Diagnosis

As for the type and only species.

*Sellamynodon zimborensis* Codrea & Şuraru, 1989 [30]

1989 *“Cadurcodon” zimborensis* Codrea & Şuraru [30]: 319–338

1996 *Lartetotherium sansaniensis*? Codrea [97]: 84–87

1998 *Lartetotherium sansaniensis*? Codrea [98]: 121–125, Fig 1

2000 *Cadurcodon zimborensis* Codrea [31]: 19–23, pl I

### Holotype

**Fig 2:**
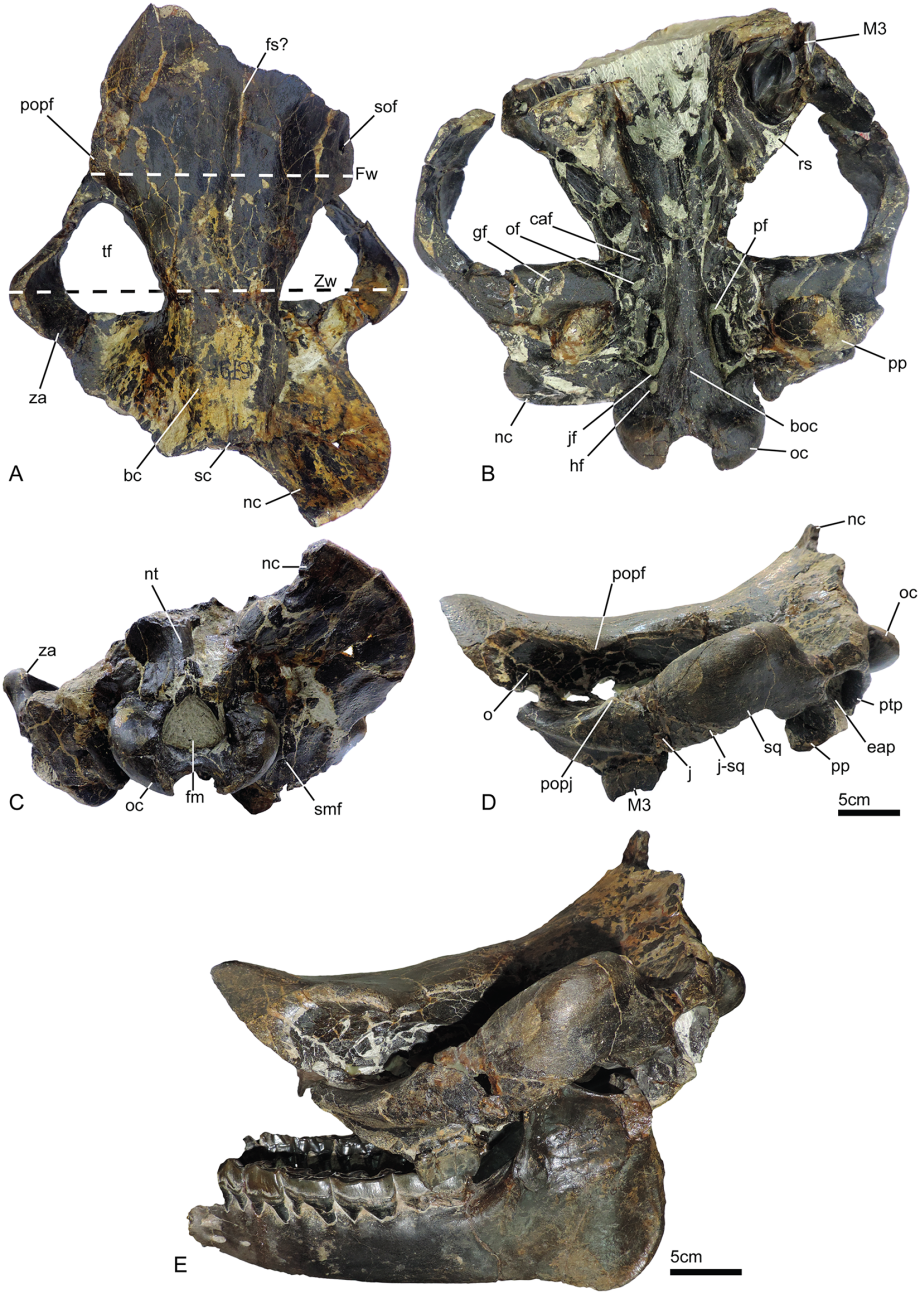
Skull of *Sellamynodon zimborensis* (holotype, UBB MPS 15795), a Late Eocene-Early Oligocene amynodontid from Dobârca (Romania). Dorsal (A), ventral (B), lateral (C) and occipital (D) views. Skull and associated mandible in lateral view (E). Abbreviations: bc, braincase; boc, basioccipital crest; caf, caudal alar foramen; eap, external auditory pseudomeatus; fm, foramen magnum; fs?, frontals suture?; Fw, frontal width; gf, glenoid fossa; hf, hypoglossal foramen; j, jugal; j-sq, jugal-squamosal suture; jf, jugular foramen; nc, nuchal crest; nt, nuchal tubercle; o, orbit; oc, occipital condyle; of, oval foramen; pf, piriform fenestra; popf, postorbital process of the frontal; popj, postorbital process of the jugal; pp, postglenoid process; ptp, posttympanic process; rs, retromolar space; sc, sagittal crest; smf, stylomastoid foramen; sof, supraorbital foramen; sq, squamosal; tf, temporal fossa; za, zygomatic arch; Zw, zygomatic width.

**Fig 3:**
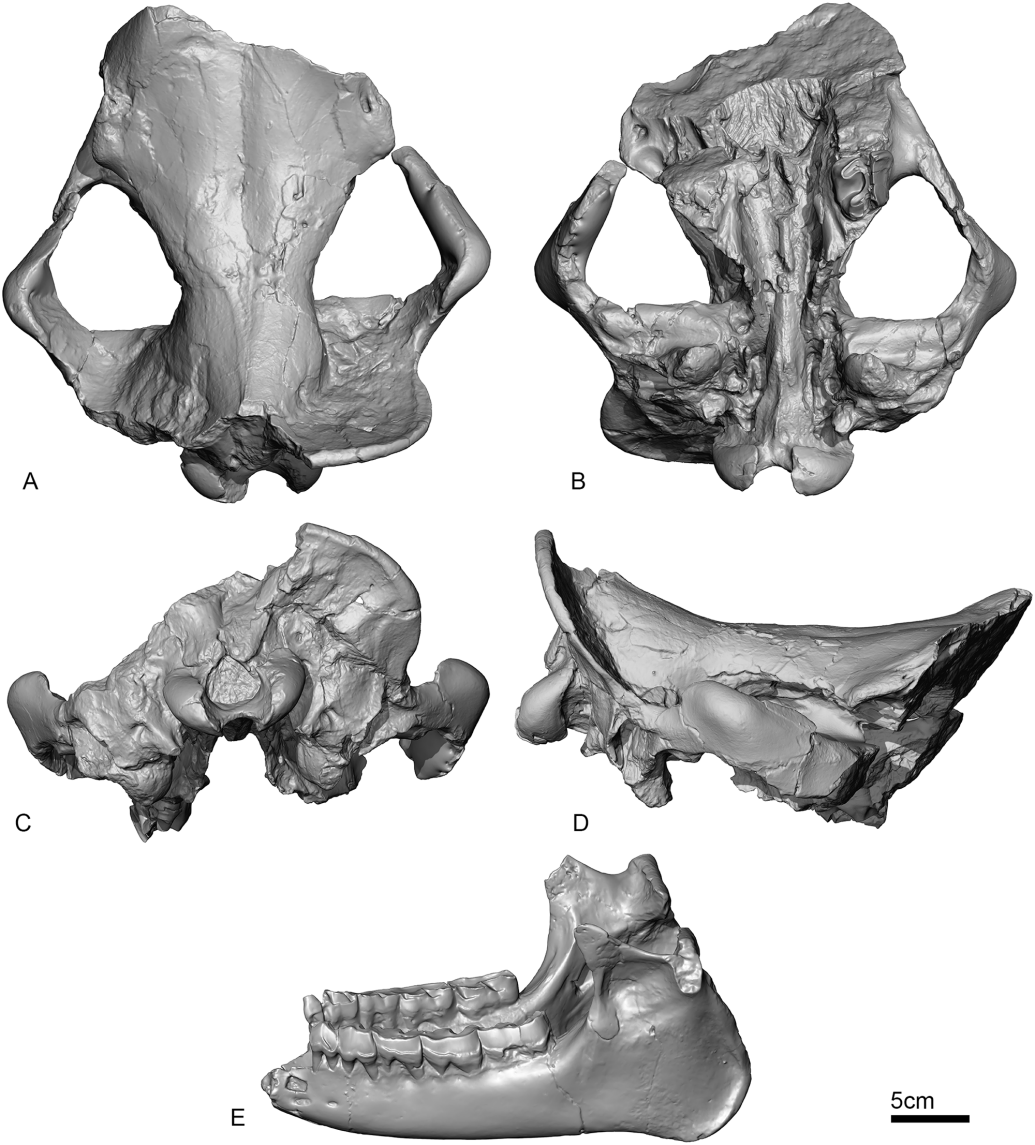
3D model in orthographic projection of UBB MPS 15795, holotype of *Sellamynodon zimborensis*. Dorsal (A), ventral (B), occipital (C) and lateral (D) views of the skull. Mandible in lateral view (E). 3D models are available at MorphoMuseuM.com [35] along with other specimens described in this publication.

**Fig 4:**
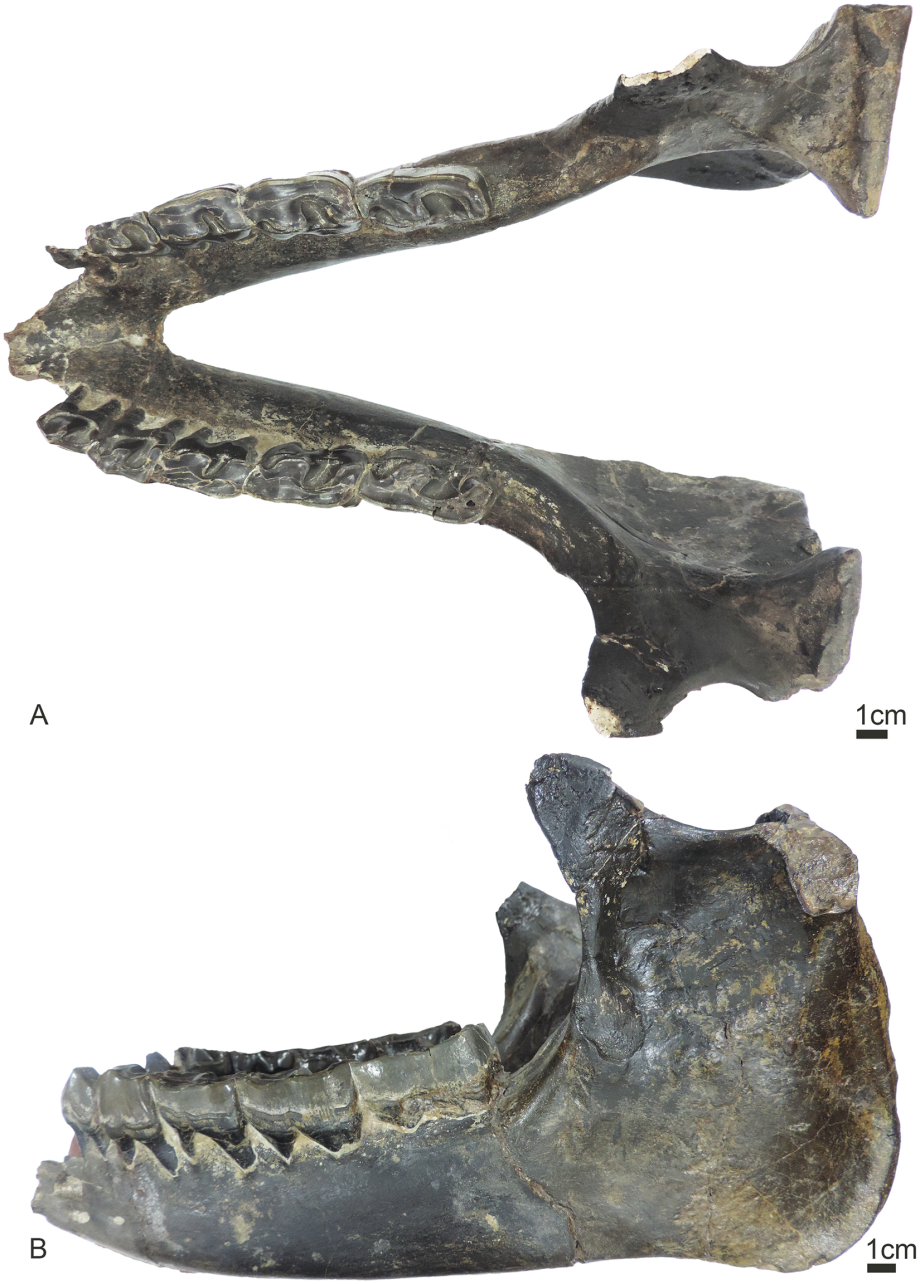
Mandible of *Sellamynodon zimborensis* (holotype, UBB MPS 15795). Occlusal (A) and lateral (B) views.

UBB MPS 15795: a skull (Figs 2 and 3A–3D) and associated mandible (Figs 3E and 4) in the Paleontology-Stratigraphy Museum collections of the UBB (Cluj-Napoca, Romania).

### Type locality and horizon

Dobârca (Sibiu County, Romania), likely Late Eocene or Early Oligocene (see Material part).

### Emended diagnosis

A medium-sized amynodontid (Lm1-3 = 114.0), distinguished from all other members of the family by the huge development of nuchal crests, a very high zygomatic arch, a deeply concave dorsal profile of the skull, the absence of a sagittal crest, the absence of a lingual groove on the body of mandible and a ramus of mandible inclined forward.

Within Metamynodontini, differs from *Paramynodon birmanicus* by the presence of a postorbital process on zygomatic arch, a right-angled anterior tip of the zygomatic process of maxilla in ventral view, an upraised mandibular symphysis, a continuous labial cingulum of lower premolars and a labial cingulum of lower molars always present. Differs from *Megalamynodon regalis* in having a right-angled anterior tip of the zygomatic process of maxilla in ventral view, a well-developed paraoccipital process, a mental foramen at the level of p2-4, a well-developed coronoid process of the mandible, a low condylar process of the mandible and a talonid of m3 enlarged. Differs from *Metamynodon planifrons* in having a high anterior base of the zygomatic process of maxilla, an upraised mandibular symphysis, a mental foramen at the level of p2-4, the presence of labial cingulum on lower premolars and molars, an enlarged talonid of m3 and a strong paracone fold on M3.

#### Remarks

*Sellamynodon zimborensis* also differs from early diverging Amynodontidae (*Rostriamynodon grangeri*, *Amynodon advenus* and *Sharamynodon mongoliensis*) by the absence of p2 and in having a smooth external groove of lower cheek teeth, an enlarged talonid on m3 and a high orbit on the skull. Further differs from *Rostriamynodon grangeri* in having an anterior border of the orbit above P4-M2, a zygomatic index (= zygomatic width / frontal width) superior to 1.5 and a mental foramen at the level of p2-4. Further differs from *Amynodon advenus* in having continuous labial cingulum on lower premolars and molars, a strong paracone fold on M3 and a well-developed paraoccipital process. Further differs from *Sharamynodon mongoliensis* by the presence of a postorbital process on zygomatic arch, an upraised mandibular symphysis and a posterior margin of the mandibular symphysis at the level of p2-4.

Differs from Cadurcodontini, in having a flat postglenoid process and a low condylar process of the mandible. Further differs from *Zaisanamynodon* in having a postorbital process on the zygomatic arch, a developed nuchal tubercle, a zygomatic index superior to 1.5, a right-angled anterior tip of the zygomatic process of maxilla in ventral view, a subtriangular foramen magnum, an upraised mandibular symphysis, a mental foramen at the level of p2-4, in having cement on upper cheek teeth, continuous labial cingulum on lower premolars, and a wide space between condylar and coronoid processes of the mandible. Further differs from *Amynodontopsis* and *Cadurcotherium* in having a zygomatic index superior to 1.5, weak or variable cement on cheek teeth, labial cingulum of lower molars always present and a high orbit on the skull. Further differs from *Cadurcodon* in having a well-developed coronoid process of the mandible, an upraised mandibular symphysis, weak or variable cement on cheek teeth, an oblique hypolophid of lower molars and a high orbit on the skull.

### Description

#### Skull (Figs 2A–2D and 3A–3D)

In dorsal view (Figs 2A and 3A), the sutures for nasals (if present) and parietals are not distinguishable but there is a distinct mesial line that could be the suture line separating the two frontals. Frontals are wide and supraorbital foramen are present. The left anterior side of the skull is more complete than the right side. On its anterolateral extremity, the skull is slightly dorsally elevated and the bone surface is coarse. The postorbital process of the frontal does not enclose the orbit, which is open into the wide temporal fossa. There is no frontoparietal or sagittal crest, only a very low ridge at the posteriormost preserved part of the skull (that could potentially give birth to a larger posterior crest) and no sign of any horn attachment. The braincase is distinguishable by the constriction of the skull. It is completely bordered posteriorly by a large bony “frill” that is formed by the extraordinary growth of the nuchal crest. It is a rather thin, almost circular, bony edge, lacking ornamentation, which arises from the posterior end of the mastoid or temporal crest. It extends at its widest part up to around eight cm from the rest of the skull, but it is broken on the left and posterior sides. The zygomatic arches are preserved, they are wide and high. The maximum width at the zygomatic (Zw) is 317.0, whereas it is 196.0 at the frontals (Fw): the zygomatic index (= Zw/Fw) is therefore of 1.6.

In ventral view (Figs 2B and 3B), the only preserved tooth is the left M3, but there are still parts of the roots for the right M3. The skull is not preserved anteriorly to this teeth. The retromolar space is small. The left zygomatic arch is complete and diverges quickly and strongly from the skull on the left side. It starts approximately above the M2 (not preserved) and is separated from the skull above the middle of the M3. The right side of the skull is slightly less complete; the anterior part of the zygomatic arch is broken posterior to its maxilla suture. The choanae open approximately at the level of M3. The vomer is rounded. The basioccipital is well-preserved and there is a sagittal crest on the basilar process. It is bordered laterally by the piriform fenestra (= lacerum foramen) and the jugular foramen. This fenestra is widely open and shaped like a ‘C’. The hypoglossal foramen is in the middle of the ventral condyloid fossa, posterior to the jugular. The caudal alar foramen is present anteriorly to the oval foramen and to the piriform fenestra. The retroarticular process (post-glenoid apophysis) is wide and flat. The articular tubercle of the squamosal is straight and rather high. The posttympanic process is poorly developed whereas the paraoccipital process is more developed and they are both fused at their base.

Viewed laterally (Figs 2C, 2E and 3C), the dorsal profile of the skull is deeply concave. The nuchal crest is oriented posterodorsally. Posteriorly, the occipital condyles are well-expanded and oriented posteroventrally. The zygomatic arch is very high and deeply convex dorsally. It is very thick dorsoventrally: approximately 6 cm at its broadest point, from ventral to dorsal side. The postorbital process is present on the jugal. The orbit is large (around 6 cm in diameter) and well-defined, and its anterior border was anterior to the M3, possibly above premolars or M1. The suture between the jugal bone and squamosal is straight and oblique. The anterior margin of the orbit is much more anterior than the M3, it may have been approximately above the M1 (not preserved). The external acoustic meatus is closed.

In occipital view (Figs 2D and 3D), the “frill” is very large (even though it is only preserved on the right side). The foramen magnum is triangular. There is a small nuchal tubercle (or occipital protuberance) on the occipital. The stylomastoid foramen is visible between the posttemporal and paraoccipital processes. The condyles are smooth, without ridge and slightly expanded ventrally. They are directed almost perpendicularly to the frill.

#### Mandible (Figs 3E and 4)

The mandible is almost complete, some parts have been reconstructed from the counterprint in the sediment. Only the anterior part of the symphysis is missing and p3-4 are incomplete on the right side. The basic dimensions are given in Codrea & Şuraru [30]. The symphysis was either slightly upraised or horizontal, and not very massive. Its posterior margin stops at the level of p4. There are several mental foramina and they are mostly located at the level of p3. The lower base of the body of mandible is straight and there is no lingual groove medially. The height of the body is almost constant. The ramus of mandible is stout and inclined forward. The coronoid process is well developed longitudinally, but very thin, and the condylar process is low. There is a wide gap between these two processes. There is no post-cotyloid process. The mandibular foramen is large and below teeth neck. The angle of the mandible is very strong, and acute. The masseteric fossa is extremely marked, forming a deep pocket in the upper part of the ramus.

The dental formula is classical for Amynodontidae, with a reduction of premolars to p3-p4, and three lower molars. The anterior dentition is unknown, though it is possible to distinguish small root remains, but it is not possible to determine if those were for canines or incisors. There is however a rather long diastema anterior to the p3, and no alveoli for p2 or p1.

#### Dentition (Fig 5; Table 2)

**Fig 5:**
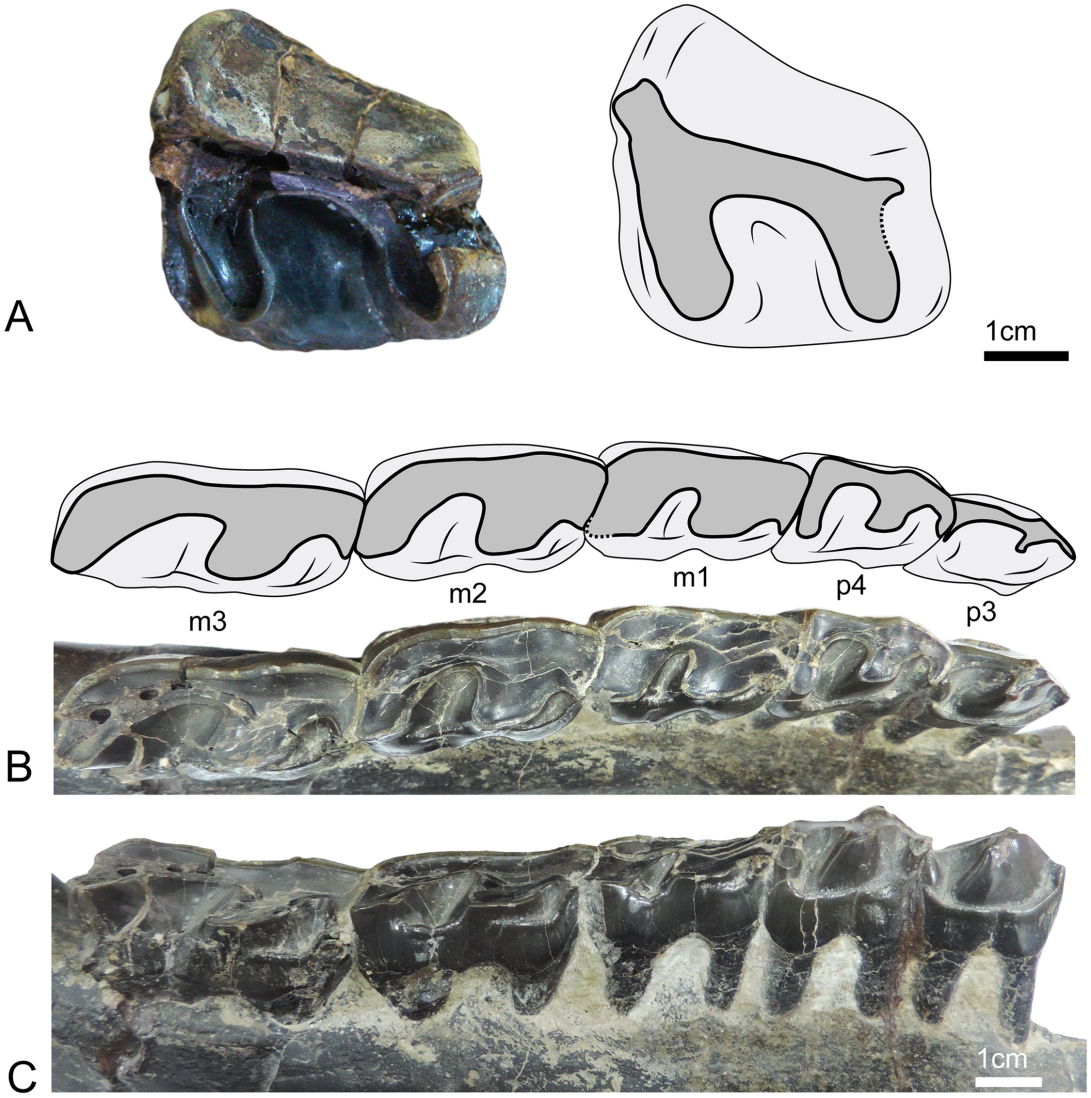
Dentition of UBB MPS 15795, holotype of *Sellamynodon zimborensis*. A: Left M3 in occlusal view. B-C: Left lower cheek teeth (p3-m3) in occlusal (B) and lingual (C) views.

**Table 2:** Measurements (in mm) of the dentition of UBB MPS 15795, holotype of *Sellamynodon zimborensis*.

*Tooth*	*Length (L)*	*Width (W)*	*Height (H)*
*M3*	41.5 / -	42.6 / -	25.0 / -
*p3*	21.0 / -	16.3 / -	20.0 / -
*p4*	25.0 / -	21.5 / -	23.0 / -
*m1*	29.0 / 28.0	19.3 / 18.4	- / -
*m2*	38.0 / 37.0	22.3 / 21.9	- / -
*m3*	47.0 / 47.0	21.6 / 21.2	- / -

The measurements are presented as left/right.

The M3 (Fig 5A) is not much worn and enamel is wrinkled. The roots are not visible. The ectoloph wall is covered in part by a very thin layer of cement. Labial and lingual cingulum are absent. The posterior cingulum is continuous. The tooth is very simple and shows no enamel folding. There is no crochet, antecrochet or crista. It is quadrangular: protoloph, metaloph and ectoloph are clearly distinct. The ectoloph is long and nearly longitudinal. Protoloph and metaloph are short and transverse. The metaloph is slightly broken posteriorly. There is no constriction of protocone or hypocone. Parastyle and metastyle are present whereas the mesostyle is absent. The paracone fold is strong and there is no metacone fold. There is a lingual groove on the protocone that disappears before the neck. The median valley is deep and V-shaped in lingual view.

Concerning lower cheek teeth (Fig 5B and 5C), p3 is distinctly shorter than p4 and premolariform, with very little developed hypoconid and entoconid, whereas p4 is almost molarifom, but still retains a developed paralophid. Talonid is much wider than trigonid on p4. Trigonid and talonid of lower molars are rounded and obtuse and the talonid of m3 is longer than trigonid. Metaconid and entoconid are never constricted. The hypolophid of lower molars is oblique, but not as sagittal as in *Cadurcotherium*. There is no lingual groove of the entoconid. The labial cingulum of lower cheek teeth (Fig 4B) is strong and continuous. The lingual cingulum (Fig 5C) is interrupted under the entoconid and metaconid of lower molars but continuous on premolars. The external groove is absent and there is no cement.

#### Remarks

The presence of a large nuchal crest on the posterolateral border of the skull seems to be a unique character among Amynodontidae. It may be homologous to the lambdoidal crest of *Zaisanamynodon* [3,89], but it is much more developed here. No other observed taxon shares such a deeply concave skull. In most species, the skull is in fact quite flat or even convex and the nuchal crest is never as developed (for instance *Metamynodon planifrons* [68] or *Amynodon advenus* [46]). This morphology is more common in Brontotheriidae, such as *Diplacodon gigan* [99] for example.

It also differs from all other Amynodontidae by the absence of a sagittal crest, or at least by its very strong reduction (it is possible that a very low sagittal crest was present on the posteriormost part of the skull, but this part is broken). The presence of a large sagittal crest was in fact considered as a diagnostic character of amynodontids [2,15]. Also, contrary to all other rhinocerotoids, the dorsolateral surface of the braincase is completely smooth because there is no parasagittal ridge. Parasagittal ridge and sagittal crest are normally used as muscle supports, which means that for *Sellamynodon* the muscles had to connect differently on the skull (on the nuchal crest for example), or were much reduced. The absence of sagittal crest could also mean that the specimen was a juvenile [21], but this hypothesis is not compatible with the presence of a fully grown and slightly worn M3. It could also be linked to sexual dimorphism, but we lack data for comparison.

The M3 though, is typically amynodontid-like. Its quadrangular shape, with its long and flat ectoloph and the presence of a metastyle are diagnostic characters of Amynodontidae [2]. In Rhinocerotidae such as *Trigonias* or *Ronzotherium* for example, the metacone is lost or fused with the paracone and the ectoloph is very short. The amynodontid M3 is more similar to the M3 of some hyracodontids such as *Hyracodon*, *Triplopus* or *Forstercooperia*, with which it shares a metastyle, though the ectoloph is shorter and more lingually deflected in these taxa. The absence of crista, crochet and antecrochet is also diagnostic of Amynodontidae [2].

Therefore, according to this unique combination of characters (absence of sagittal crest, great development of the nuchal crest, quadratic M3, reduction of lower premolars to two and elongation of talonid) this specimen represents a new genus of amynodontid, as was suspected when the mandible of *Sellamynodon zimborensis* was first described [30].

The body mass of *Sellamynodon zimborensis* was estimated following the regression equation [m = exp(1,5133 x ln(L x W) + 3,6515] of Perissodactyla [100]. The body mass (m) is in grams, L and W are length and width of m1 in mm, respectively. The first lower molar (m1) was proposed as the most suitable for this estimation, due to its low variability. With L = 28.5mm and W = 19.6mm (see Table 2 in [30]), the body mass of *S*. *zimborensis* is estimated to be around 550kg. Recently, the same equation was used for 18 amynodontid species [4] and it appears that the bodymass of *S*. *zimborensis* is very close to the most primitive amynodontid, *Rostriamynodon grangeri* (535kg). It is also similar to some specimens of *Amynodon advenus* and *Amynodontopsis bodei* (S2 in [4]). We also used a similar equation based on the ectoloph length of M3, which gives an even smaller body mass, of approximately 410kg.

Cadurcodontini Wall, 1982 [101]

*Amynodontopsis* Stock, 1933 [49]

*Amynodontopsis* aff. *bodei*

### Referred material

**Fig 6:**
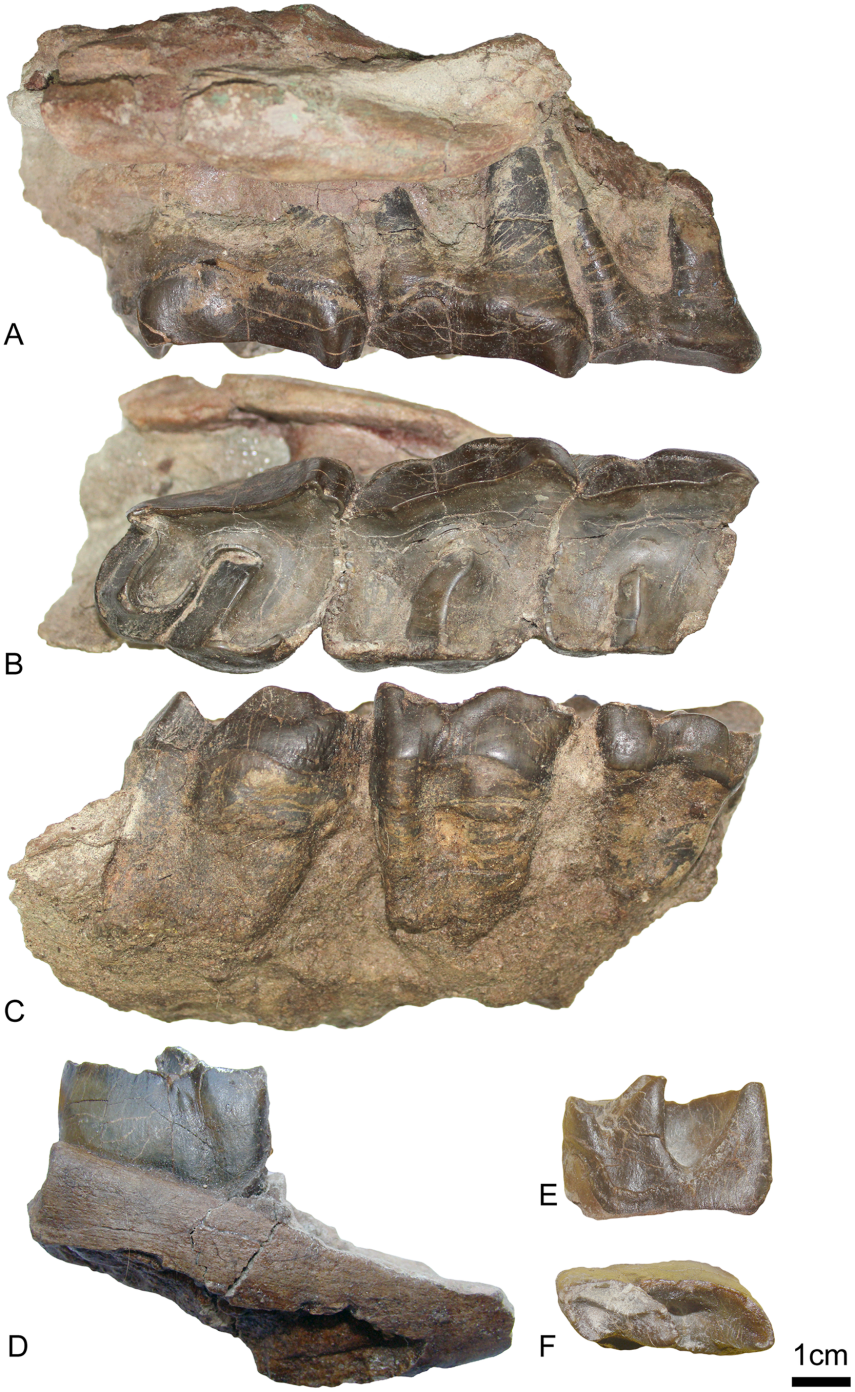
*Amynodontopsis* aff. *bodei* from Morlaca (Late Eocene; Romania). Right maxillary (UBB MPS V545) with M1-3 in labial (A), occlusal (B) and lingual (C) views. D: Right mandibular fragment from Morlaca (UBB MPS V546) with m1/2, in labial view. E: Lower right m1/2 from Morlaca (UBB MPS V546) in labial view. F: Lower right m1/2 from Morlaca (UBB MPS V546) in occlusal view.

**Fig 7:**
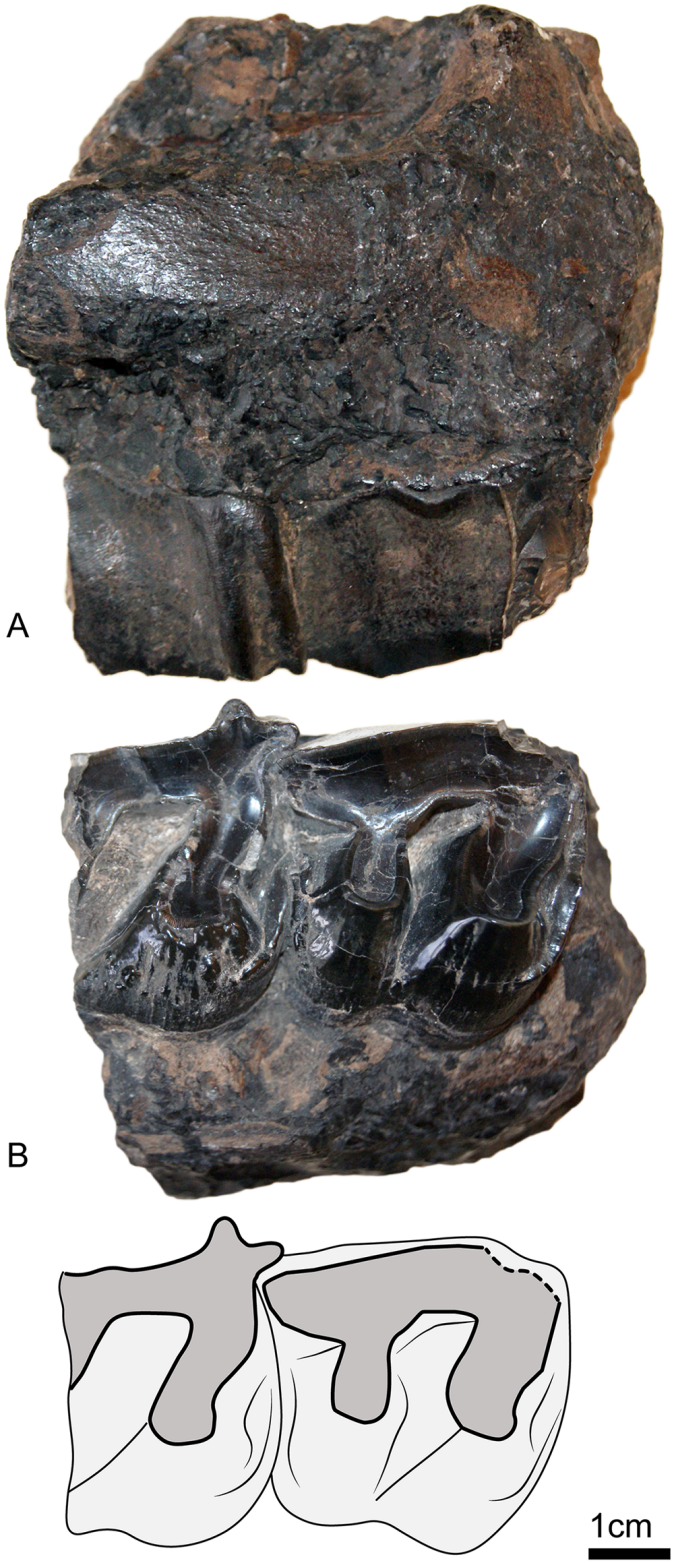
*Amynodontopsis* aff. *bodei* from Dorog (late Middle Eocene; Hungary). A-B: Right maxillary fragment (HNHM PAL 2017.54.1) with upper M2-3 in labial (A) and occlusal (B) views.

A right maxillary fragment (UBB MPS V545) with M1-3 (Fig 6A–6C) and a mandibular fragment (UBB MPS V546) with m1/2 (Fig 6D–6F). Both specimens are from Valea Nadăşului Formation [26] in Morlaca (Cluj district, Romania), a Late Eocene locality. They are stored in the Museum of Paleontology-Stratigraphy of the UBB. A right maxillary fragment (HNHM PAL 2017.54.1) with broken M2-3 (Fig 7) from Dorog Coal Formation in Dorog Basin (Hungary), a Middle Eocene locality, stored in the HNHM.

### Description

#### Maxillary (Figs 6A–6C and 7)

The zygomatic arch (Fig 6A) is slender and does not deviate strongly from the maxilla. It starts above M2 and is high above the teeth crowns. There is a ventral groove between the zygomatic and the teeth on the specimen from Morlaca, not visible on the specimen from Dorog.

#### Dentition (Figs 6 and 7; Table 3)

**Table 3:** Measurements (in mm) of the dentition of UBB MPS V545, UBB MPS V546 and HNHM PAL 2017.54.1, referred to *Amynodontopsis* aff. *bodei*.

*Localities*	*Tooth*	*Length (L)*	*Width (W)*	*Height (H)*
*Dorog (HNHM PAL 2017*.*54*.*1)*	M2	(37.5)	(40.1)	24.4
*Dorog (HNHM PAL 2017*.*54*.*1)*	M3	>24.7	40.7	25.8
*Morlaca (UBB MPS V545)*	M1	32.6	38.8	
*Morlaca (UBB MPS V545)*	M2	45.0	42.0	
*Morlaca (UBB MPS V545)*	M3	44.4	43.0	
*Morlaca (UBB MPS V546)*	m1/2	37.5	18.5	21.0

The roots of upper molars (clearly visible on UBB MPS V545; Fig 6A and 6C) are fused on the lingual side and separated on the labial side. There is no cement. Lingual cingulum is only slightly present under the protocone of M1 and the hypocone of M3. Labial cingulum is present and continuous but very thin and weak. The median valley is narrow, acute and V-shaped. The protocone is slightly anteriorly constricted on HNHM PAL 2017.54.1. There is a distinctive oblique lingual blade joining the base of the hypocone to the top of the protocone (Figs 6B, 7B and 7C). Because of this blade, the postero-lingual edge of the protocone is sharpened and not rounded. There is no crochet, antecrochet or crista. The medifossette is always absent. Metaloph and protoloph are transverse. The paracone fold is present and it is strong on the M3 (Figs 6A, 6B, 7B and 7C). There is no metacone fold. A metastyle is present on the M3 and it is large on the M2. The M1 is the smallest tooth of the row. The hypocone of the quadratic M3 is much more labial than on the other molars, and the metaloph much shorter.

The lower molar (probably m1 or m2; Fig 6D–6F) belongs to a young individual, it is almost not worn. The ectolophid groove separating trigonid and talonid is present and vanishing before the neck. The talonid is much wider than the trigonid. The trigonid is rounded and obtuse and the hypolophid is oblique. Metaconid and entoconid are not constricted. Lingual cingulum is present and reduced and labial cingulum is present anteriorly and may have been continuous, but only a small part is visible since the tooth is not fully erupted.

#### Remarks

The upper molars of the specimens from Morlaca and Dorog share all the diagnostic characteristics of the Amynodontidae: absence of crista, crochet and antecrochet, parastyle reduced and quadratic M3 with large metastyle and labial metacone [2].

Several characteristics differ from those of the skull of *Sellamynodon zimborensis*. Cement is completely absent on upper molars, the metastyle is directed postero-labially and the lingual blade joining the protocone to the base of the hypocone is present on the specimens from Morlaca and Dorog. Furthermore, even though it is incomplete, the zygomatic arch is not as strong as in *S*. *zimborensis*. In ventral view, it is also less deviated from the maxilla than in *S*. *zimborensis* and a ventral groove is present. The lower molar also differs from *S*. *zimborensis* by a greater development of the external groove of the ectolophid and by a greater width of the talonid compared to the trigonid.

Within Cadurcodontini, it differs from *Cadurcodon* by a weak paracone fold on M1-2 and from *Cadurcotherium* by the presence of lingual and labial cingulum on upper molars and an oblique hypolophid on lower molars. It also differs from *Zaisanamynodon* by a shorter metastyle on M1-2 and the absence of a postfossette on M1. It shares with *Amynodontopsis* a moderate angle of the zygomatic process of the maxilla in ventral view, the presence of an external groove on lower cheek teeth, a weak paracone fold on M3 and the absence of cement on upper teeth, but differs from *A*. *bodei* by a higher anterior base of the zygomatic process of the maxilla and a metasyle of M3 directed more posterolingually. It is therefore tentatively assigned to *Amynodontopsis* aff. *bodei*. Its body weight is estimated to be around 500kg with the equation based on the length of M3, which is close to the previously estimated body weight of *Amynodontopsis bodei* (S2 in [4]).

## Phylogenetic relationships

**Fig 8:**
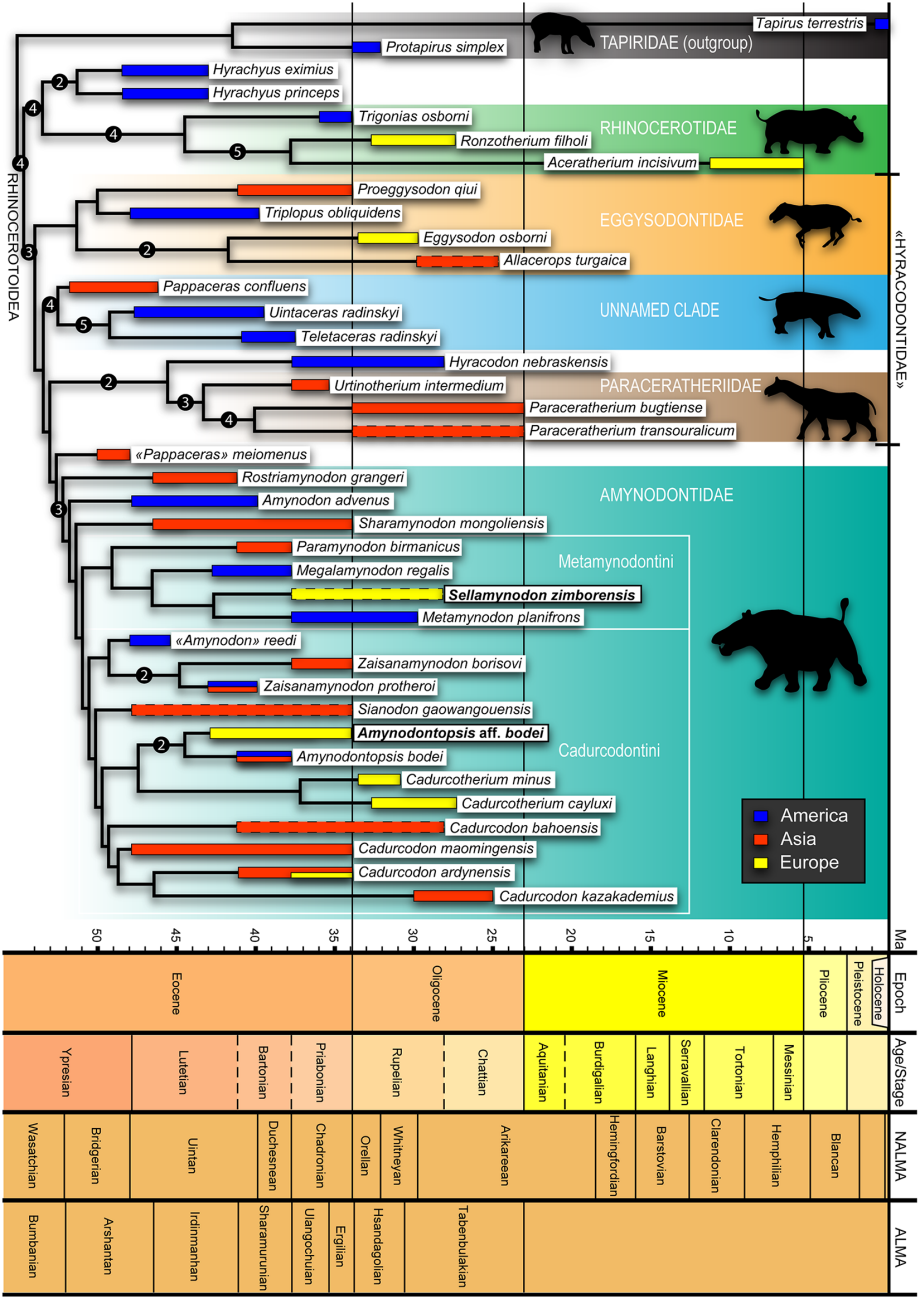
The single most parsimonious tree and the distribution of rhinocerotoids (excluding uninformative characters), scaled in time. Tree length = 825, CI = 0.30, HI = 0.70, RI = 0.51 and RC = 0.15. Taxa ages based on literature as listed in Table 1. Thick bars represent temporal and spatial distribution of taxa and thick bars with dashed lines have uncertain ages. Geological time scale produced with TSCreator [103]. Numbers at nodes are Bremer support values.

One most-parsimonious tree of 825 steps (excluding uninformative characters; CI = 0.30, HI = 0.70, RI = 0.51 and RC = 0.15; Fig 8) was retrieved during the heuristic search in PAUP* version 4.0a159 and PAUP* version 4.0b10. The same topology was found with the traditional search in TNT version 1.1. It should be noted that PAUP* version 4.0b10 was unable to show unambiguous synapomorphies in ACCTRAN optimization, whereas PAUP* version 4.0a159 depicted them accurately. This version was then used to visualize transformations, as well as TNT and Mesquite version 3.2 [102]. The complete list of transformations is provided in S2 File.

Suprageneric relationships within Rhinocerotoidea are divergent from other recent phylogenies, especially concerning the Hyracodontidae, which is clearly paraphyletic (following the “classical” definition of the family), as also recovered by recent study [23]. The results presented here are disputable for several reasons. Firstly, the taxonomic sampling is unequal depending of the families (it is almost exhaustive for Amynodontidae, but poor for Rhinocerotidae and Hyracodontidae). Secondly, our matrix was not designed to resolve the phylogeny of early rhinocerotoids. Phylogenetic results outside Amynodontidae presented here are thus preliminary, but this analysis is necessary to discuss their position within Rhinocerotoidea. Indeed, it was previously investigated only through the use of two basal amynodontid taxa [20–23], which is clearly insufficient to represent the variability of this group.

Most nodes have very low support values, *i*.*e*. very few clades are supported with a Bremer support value superior to 1. The best supported clades are basal to Amynodontidae, with Bremer values of 5: the clade *Aceratherium*-*Ronzotherium* and the clade *Uintaceras*-*Teletaceras*. Amynodontidae have a Bremer support value of 3 and within Amynodontidae, only two clades have support values higher than 1: *Zaisanamynodon* and the clade *Amynodontopsis* aff. *bodei*-*Amynodontopsis bodei*. These low support values indicate that taxonomic inferences should be taken cautiously. They are probably due to a small taxonomic sampling of Rhinocerotidae, for which the original characters matrix was designed [36,37], compared to an almost exhaustive sampling of Amynodontidae.

### Rhinocerotidae

Rhinocerotidae *sensu stricto* (*Trigonias*, *Ronzotherium* and *Aceratherium*) is supported by 11 unambiguous synapomorphies, including the presence of tusk-like i2 (*79*), the absence of i3 (*81*) and of lower canine (*82*). They are supported by a Bremer value of 4. Contrary to most previous studies [20–23,104], the monophyletic *Hyrachyus* is here closer to Rhinocerotidae than to all other rhinocerotoids. However, our taxonomic sampling of Rhinocerotidae is very small, so the proposed relationships are only tentative and should be further investigated in future studies. Yet, *Hyrachyus* and Rhinocerotidae indeed share seven synapomorphies whose placement is unambiguous: hypocone and metacone of P4 separated (*108*), lingual cingulum of lower premolars usually absent (*147*), mesostyle of D2 present (*165*), lateral vertebral foramen of the atlas absent (*185*), indentation on the medial side of the magnum present (*218*), antero-distal groove of the tibia present (*242*) and latero-distal gutter of the fibula shallow (*250*).

### “Hyracodontidae”

Seven unambiguous synapomorphies support the smallest clade including *Amynodon* and *Eggysodon* (Bremer support = 3): the mandibular symphysis is massive (*42*), the constriction of the metaloph is present on P2-4 (*86*), the metaloph of P2 is transverse (*95*), the metastyle of M1-2 is long (*120*), the hypolophid of lower molars is oblique (*161*), the ectolophid fold of d2-3 is absent (*177*) and the metapodials intermediate relief is low and smooth (*280*). According to our results, “Hyracodontidae” are basal to Amynodontidae and are clearly paraphyletic. The monophyly of Hyracodontidae has long been debated, but was hardly ever found by computed phylogenetic analyses [20,21], and it can in fact be considered as a wastebasket taxon [105]. Recently, this group has been redefined as the most basal clade of rhinocerotoids that includes *Triplopus* and *Hyracodon* [23], but we did not find this clade either. In our tree, *Triplopus* is found as sister-group of *Proeggysodon* and *Hyracodon* as sister-group of Paraceratheriidae (Bremer support value of 2). In our analysis, “Hyracodontidae” can be divided into three clades: Eggysodontidae, Paraceratheriidae and an unnamed clade. Eggysodontidae is the most basal of them and shows two dichotomies: *Eggysodon*-*Allacerops* and *Proeggysodon*-*Triplopus*. This clade is supported by three unambiguous synapomorphies: P3-4 protocone and hypocone connected by a lingual bridge (*102*), lower molars lingual cingulum always present (*157*) and proximal border of the anterior side of magnum concave (*217*). Within Eggysodontidae, *Eggysodon* and *Allacerops* share seven unambiguous synapomorphies and are supported by a Bremer value of 2.

The next most basal clade (“unnamed clade”) includes *Pappaceras confluens* and two equivocal rhinocerotids: *Uintaceras radinskyi* and *Teletaceras radinskyi*. It is quite strongly supported by a Bremer value of 4. *Teletaceras* was classically considered as a primitive Rhinocerotidae while *Uintaceras* was often considered as the closest sister-group to this family [22,106,107]. Yet, according to our results they both branch outside of the Rhinocerotidae. Though it is not quite surprising for *Uintaceras* (a similar position far from Rhinocerotidae was also recently recovered [23]), it is more startling for *Teletaceras*, who shares with Rhinocerotidae the I1/i2 tusk complex, though it is less developed than in true rhinocerotids [107]. However, they do not share the absence of lower canine. Again, this may be an effect of our small taxonomic sampling of Rhinocerotidae. As previously explained, taxonomical results discussed here are tentative. Nonetheless, the union of *Uintaceras* and *Teletaceras* is strongly supported, with a Bremer support value of 5, and they share four unambiguous synapomorphies, including the reduction of lower canine (*284*). They both share six unambiguous synapomorphies with *Pappaceras confluens*: lateral apophysis of nasal present (*1*), P2-4 postfossette narrow (*89*), upper molars labial cingulum always present (*109*), lower molars labial cingulum reduced (*150*), p2 paralophid isolated (*154*) and lower molars labial cingulum usually present (*159*).

Furthermore, we did not find any close relationship between *Pappaceras* (*P*. *confluens* or “*P*.*” meiomenus*) and Paraceratheriidae. We did find a monophyletic Paraceratheriidae clade with a Bremer support value of 3, including *Paraceratherium* and *Urtinotherium*, supported by three unambiguous synapomorphies: mandibular symphysis nearly horizontal (*53*), ectoloph and metaloph of M3 fused [= ectometaloph; (*133*)] and Cc 2 and Cc 3 facets of the astragal always independent (*263*). This clade was found as sister-group of *Hyracodon nebraskensis*, with whom it shares six unambiguous synapomorphies: sphenorbital foramen and foramen rotundum are fused (*16*), contrary to “*P*.*” meiomenus* where they are “probably separated by a thin plate” [23], protocone and hypocone of P2 are separated (*94*), P3-4 protocone and hypocone are connected by a lingual bridge (*102*), upper molars antecrochet is usually present (*110*), upper molars lingual cingulum is always absent (*114*) (it is always present on “*P*.*” meiomenus*) and lower canine is reduced (*284*) (even absent in *Paraceratherium*). It reveals that Forstercooperiinae and Paraceratheriidae (*sensu* [23]) may not be monophyletic: “*P*.*” meiomenus* is closer to amynodonts than Paraceratheriidae, whereas *P*. *confluens* is closer to *Uintaceras* and *Teletaceras* than to Paraceratheriidae. However, our results must be compared with caution considering that the species used for our analysis differ from their analysis. A comprehensive revision of this group, which is beyond the scope of our study, is needed.

### Amynodontidae

*“Pappaceras” meiomenus* was recently described as a forstercooperine and the “earliest known unequivocal rhinocerotoid” [23]. Interestingly, it was stated that Forstercooperiinae was the sister clade of Paraceratheriinae, and that both formed a clade (Paraceratheriidae), sister to the Amynodontidae. The Forstercooperiinae clade was united by two synapomorphies: nasal notch above canines and M1-2 metaloph short (S1 in [23]), but we did not find the monophyly of this clade with our matrix. However, based on their results (Fig 4 in [23]), the genus *Pappaceras* is not monophyletic, as it includes *Forstercooperia totadentata*. “*P*.*” meiomenus* should rather be included in a new genus, or alternatively, *Pappaceras* should be a junior synonym of *Forstercooperia*. In any case, with an Arshantan age *Pappaceras* remains the earliest known unequivocal rhinocerotoid.

In contrast, our results suggest that “*P*.*” meiomenus* could be considered as one of the first amynodontid. We have found indeed that it shares with them three unambiguous synapomorphies: the premolar/molar series is very short (length of P3-4 / length of M1-3 < 0.42, (*63*); it is here equal to 0.38), upper P1 is sometimes absent (*91*) and M3 metastyle is directed posterolabially (*297*). The close resemblance of “*P*.*” meiomenus* with *Rostriamynodon* or other Amynodontidae was also previously noticed [23], as they share “a wide frontal between the orbital and followed by a flange overhanging the postorbital cavity”, “incisors arranged in straight lines and weakly converging anteriorly”, “ridge-like nuchal crest”, “a relatively large canine, distinctly reduced P1, a short postcanine diastema, P4 transversely wide with high and strong protoloph, weak and short metaloph (metaconules), and M3 quadrate in outline” [23]. In particular, the reduction of P1 and the distinctly quadrate M3 in addition to its labially deflected metacone strongly suggest an amynodontid, and very much differ from other *Pappaceras* or Paraceratheridae. In *Pappaceras confluens* for example (Fig 1 in [69]), the upper P1 is always present and two-rooted (whereas if present, it is one-rooted in “*P*.*” meiomenus*), a primitive state, and the ectoloph and metaloph of the triangular M3 are almost fused (i.e. the metacone is not lingually deflected). Finally, it also shares a “distinct preorbital fossa” [23], which is a characteristic of Amynodontidae, even though it may also be present in *Forstercooperia* or *Paraceratherium*. In *Rostriamynodon* as in “*P*.*” meiomenus* the maxilla is excluded from the border of the nares, due to the broad extension of the premaxilla. The relatively large size of the canines is also characteristic of Amynodontidae, as well as the long metastyle of the M3.

The monophyly of Amynodontidae is retrieved and *Rostriamynodon grangeri*, *Amynodon avenus* and *Sharamynodon mongoliensis* are placed as early diverging amynodontids, but not *Amynodontopsis*, contrary to recent results [4]. The clade Amynodontidae is supported by a Bremer support value of 3. The monophyly of Amynodontidae (defined here as the smallest clade including *Rostriamynodon* and *Cadurcodon*) is supported by six synapomorphies whose placement is unambiguous: lacrimal process is absent (*8*), anterior base of the zygomatic process of the maxilla is high (*10*), rostral end of nasal bones is broad (*24*), P1 in adults is always absent (*91*), upper canine is strong (*283*) and upper molars parastyle is reduced (*296*).

Two clades previously defined [4] are identified: Metamynodontini and Cadurcodontini. The recent definition of Metamynodontini is the “stem-based taxon that includes *Metamynodon planifrons* and all amynodontids closer to it than to *Cadurcodon ardynensis*”. Following this definition, *Sellamynodon zimborensis* is included in this clade, as well as *Paramynodon* and *Megalamynodon*. *S*. *zimborensis* is found as sister-group of *Metamynodon planifrons*, based on the sharing of one unambiguous synapomorphy: the external auditory pseudomeatus is closed (*18*). *Megalamynodon*, *Metamynodon* and *Sellamynodon zimborensis* are in a same clade and share three unambiguous synapomorphies: protocone and hypocone of P3 are separated (*102*), upper molars crista is usually absent (*112*) and upper postcanine diastema is short (*291*). Metamynodontini is supported by four unambiguous synapomorphies: zygomatic arch is high (*11*), postglenoid process of squamosal is flat (*42*), upper molars antecrochet is always absent (*110*) and the humerus distal articulation has a deep median constriction (diabolo-shaped; *194*).

The other clade, Cadurcodontini, was recently defined as the “stem-based taxon that includes *Cadurcodon ardynensis* and all amynodontids closer to it than to *Metamynodon planifrons*” [4]. It is here supported by six unambiguous synapomorphies: the nasal notch is above P4-M1 (*3*), the postglenoid process of the squamosal is dihedral (*42*), the M1-2 paracone fold is weak (*118*), the olecranon fossa of the humerus is low (*193*), the talonid of m3 is longer than trigonid (*288*) and the diastema between upper incisors and canine is absent (*290*). *Cadurcodon*, *Cadurcotherium* and *Zaisanamynodon* are all monophyletic. *Amynodon reedi* Stock, 1939 was described based on a skull fragment with P4-M2, from the Late Eocene of California [48]. According to our phylogenetic results, it is not found close to *Amynodon advenus* (the type species of *Amynodon*) at all, but instead as sister-group of *Zaisanamyodon*. Indeed, it shares one unambiguous synapomorphy with this genus (upper molars lingual cingulum usually absent; *114*) and only differs from it by one autapomorphy (the hypocone is anterior to metacone on P3-4; *103*). Except in size difference, the morphology of the teeth is very similar to this genus, much more than to *Amynodon*. For instance, contrary to *A*. *advenus*, the lingual cingulum of P4 is complete, the M1 paracone fold is also poorly marked, and the M3 parastyle is more pronounced, as in *Zaisanamynodon*. The M1 metastyle is much shorter, as in *Z*. *borisovi* and *Z*. *protheroi* and the metaloph of P4 does not connect to the protocone as in *Z*. *borisovi*. Therefore, we suggest to assign this species to *“Amynodon” reedi* until its material is re-examined. *Sianodon gaowangouensis* is found as sister group to the clade including *Amynodontopsis*, *Cadurcodon* and *Cadurcotherium*. Here, within *Cadurcodon*, *C*. *bahoensis* is more basal than *C*. *maomingensis*, contrary to recent results [4]. The sister group of *Cadurcodon* includes *Cadurcotherium*, *Amynodontopsis bodei* and the specimens from Morlaca and Dorog (scored under one same taxon: *Amynodontopsis* aff. *bodei*), that are found in our phylogenetic analysis as sister-group of *Amynodontopsis bodei*. They are united with a Bremer support value of 2 and by four unambiguous synapomorphies: the anterior tip of the zygomatic process does not strongly deviate from the maxilla (*37*), the upper molars labial cingulum is always present (*109*), the external groove of lower cheek teeth is developed (*140*) and the M3 paracone fold is weak (*289*). *Amynodontopsis* aff. *bodei* has one autapomorphy: the M3 metastyle is directed posterolingually (*297*). Here, *Amynodontopsis* and *Amynodontopsis* aff. *bodei* are placed as sister-group of *Cadurcotherium*, with which they share four unambiguous synapomorphies: the rostral end of nasal bones is narrow (*24*), the mandibular ramus is inclined backward (*60*), the upper molars antecrochet is always absent (*110*) and the lower molars labial cingulum is always absent (*159*).

## Discussion

### Phylogeny

From a phylogenetic point of view, and with the current taxonomic sampling, the monophyly of “Hyracodontidae” is seriously questioned. We confirm indeed that they could be divided in three monophyletic clades: Eggysodontidae, Paraceratheriidae and another unnamed clade including *Pappaceras confluens* and equivocal rhinocerotids like *Uintaceras* and *Teletaceras*. It is interesting to note that in these three clades, some forms show strong convergence with Rhinocerotidae. For instance, *Teletaceras radinskyi* possesses the I1/i2 chisel-tusk complex (*sensu* [108]) of rhinocerotids, though not associated to the loss of I3/i3 and canines [107], but its position in our tree may be an artifact of our taxonomic sampling and is thus tentative. Similarly, *Paraceratherium* shows the characteristic reduction of the anterior dentition by the loss of I3-C and i3-c, but without sharing the strictly I1/i2 complex, materialized instead by I1/i1 [88]. However, comparing our results with those of Wang et al. [23], we did not find the monophyletic clade including *Hyracodon* and *Triplopus*, that they named Hyracodontidae.

Furthermore, Wang et al. [23] recently described *Pappaceras meiomenus* as a forstercooperiine paraceratheriid, whereas our results suggest that it could be in fact the closest sister group of Amynodontidae. In any case, the authors had also reported the close resemblance of this form with *Rostriamynodon*, an early diverging Amynodontidae. Amynodontidae are without much doubt monophyletic and can be easily diagnosed by a number of synapomorphies (reduction of premolars row length compared to molars [also shared with *P*. *meiomenus*], loss of upper P1, M3 metacone labially deflected and strong canines). The association of these characters with a deep preorbital fossa is also possibly diagnostic. All these characters are very derived compared to those of “Hyracodontidae”.

Here, our results clearly differ from the most recent and comprehensive phylogenetic study on Amynodontidae [4] by the position of the genus *Amynodontopsis*. We show that it is much more derived than previously recovered [4], as it is indeed sister-group of *Cadurcotherium* (Fig 8). *Cadurcotherium* probably shows the most derived characters in Amynodontidae with cemented, hypsodont and very laterally-compressed cheek teeth. Although *Amynodontopsis* clearly does not show the same degree of derived character states as *Cadurcotherium*, it notably shares with it a very similar development of the preorbital fossa [15,49]. This fossa, associated to very short nasal bones and retracted narial notch, is a strong evidence for the existence of a proboscis [109], such as found in *Tapirus*, which is used to manipulate food. The similarities of this facial structure between *Cadurcodon* and *Amynodontopsis* (which were grouped together in a group named “cadurcodonts”) was already noted [109]. However, it was suggested that *Cadurcotherium* could belong to a different group [109], the metamynodonts, but its skull was unknown at this time. Contrary to Wall [2,109], and as previously suggested [15], *Cadurcotherium* belongs in fact to the same group as *Cadurcodon* and *Amynodontopsis*. It probably possessed a proboscis, like the other members of this group. Furthermore, the clade *Cadurcotherium*-*Amynodontopsis* shows a very similar development of the proboscis, as suggested by the reduction of nasal incision to the front of M1, the extension of the preorbital fossa medial to the orbit in dorsal view, a short diastema, and a reduced preorbital portion of the skull [15]. These characters indicate the presence of a well-developed proboscis, but not as developed as in *Cadurcodon* [15,109]. However *Amynodontopsis* differs from *Cadurcotherium* by its anterior dental formula: it is complete in *Amynodontopsis* (3I/3i) (though the incisors were very small), but reduced in *Cadurcotherium cayluxi* (2I/1i), which may indicate two different feeding habits, as also indicated by the differences in hypsodonty and cementation of cheek teeth. *Amynodontopsis*, with its complete incisors formula shows an identical condition to *Tapirus* for example.

### Palaeobiogeography

From a European context of mammalian history, the record of Amynodontidae in the Eocene of South Alpine-Carpathian Europe, as opposed to their absence in the rest of Europe, allows to propose a new picture of mammalian interchanges in Eurasia and improves the understanding of their diversification during the Eocene-Oligocene transition. Due to high sea-level stand of the Middle and Late Eocene, Europe was disconnected from the main continental landmasses, producing its own endemic fauna [110]. Europe had become an archipelago in a warm, tropical sea, isolated from North America by the complete opening of the Atlantic Ocean, and from Asia by the Turgaï Strait, which connected Arctic Ocean to the Tethys Sea [111,112]. After the Eocene-Oligocene transition, a fall in sea-level related to the “Oi-1 glaciation” (ca. 32 Ma; [111]) had notably caused the drying-up of the Turgaï Strait, thereby establishing a land connection with Asia. The effects in Western Europe are assumed to be extinctions and dispersal-generated originations of the Grande Coupure [17,113,114]. Besides, numerous authors have suggested that climate change due to the Oi-1 glaciation combined with competition produced this faunal turnover (e.g. [18,100,115]). The latter marked a change from the endemic European faunas to ones with major components of Asian origin, such as Rhinocerotoidea (e.g. [18,100,114,116–118]). The scarce records of Eocene European Rhinocerotoids were limited to the Early-Middle Eocene cosmopolitan *Hyrachyus* [90,119], and two Amynodontidae, assumed to be Late Eocene, *Amynodon hungaricus* (Tápiószele?, Hungary; [12]) and *Cadurcodon ardynensis* (Nikolaevo and Kameno, Bulgaria; [11]). Concerning *Hyrachyus*, its presence in Europe seems to be related to the Early Eocene palaeogeographical context during which Europe was still connected to North-America by the DeGeer Route [120,121]. Moreover, the inclusion of this genus within Rhinocerotoidea is still debated [23,122], though according to the results of our phylogenetic analysis, *Hyrachyus* could be considered as sister group of Rhinocerotidae (Fig 8). Consequently, the report here of Amynodontidae in the Eocene of Hungary and Romania is truly exceptional, considering the scarcity of Eocene rhinocerotoids in Europe. The discovery of *Sellamynodon zimborensis* in Dobârca (Romania) is also of great importance for the study of European faunas in the context of the Grande Coupure event. It represents indeed the only European species of the Metamynodontini tribe, which was previously exclusively restricted to North America (*Megalamynodon* and *Metamynodon*) and Asia (*Paramynodon birmanicus*, in Myanmar). However, the age of *S*. *zimborensis* still needs to be clarified: it could either be Late Eocene or Early Oligocene. If the age of Dobârca is Early Oligocene, then this new taxon represents the only occurrence at that time in Europe of an amynodontid other than the well-known *Cadurcotherium*, whose European distribution is restricted to France (e.g. [14,15]) and Switzerland [16] during the Early Oligocene, but extended to Spain [123] and Bosnia and Herzegovina [124] during the Late Oligocene. It is also known in Pakistan during the entire Oligocene [9]. Otherwise, if the age of the Dobârca locality is Late Eocene, then it documents a new early occurrence of Amynodontidae in Europe, which means that they were regionally more diversified than what was previously suggested by the fossil record. Furthermore, with the report of *Amynodontopsis* aff. *bodei* in Dorog (late Middle Eocene, Hungary) and in Morlaca (Late Eocene, Romania), we document the first record of the widespread *Amynodontopsis* out of Asia and North America, and also the earliest occurrence of Amynodontidae in Europe. To the best of our knowledge, *Amynodontopsis bodei* was only reported in the USA [6,48,49,125], Mexico (Chadronian age; [126]) and China (Ulangochuian age; [2,109,127]). Another enigmatic species, *Amynodontopsis parvidens*, is also reported from Urtyn Obo (Ulangochuian age; China; [127]: table 27.1) but no description of this species can be found.

**Fig 9:**
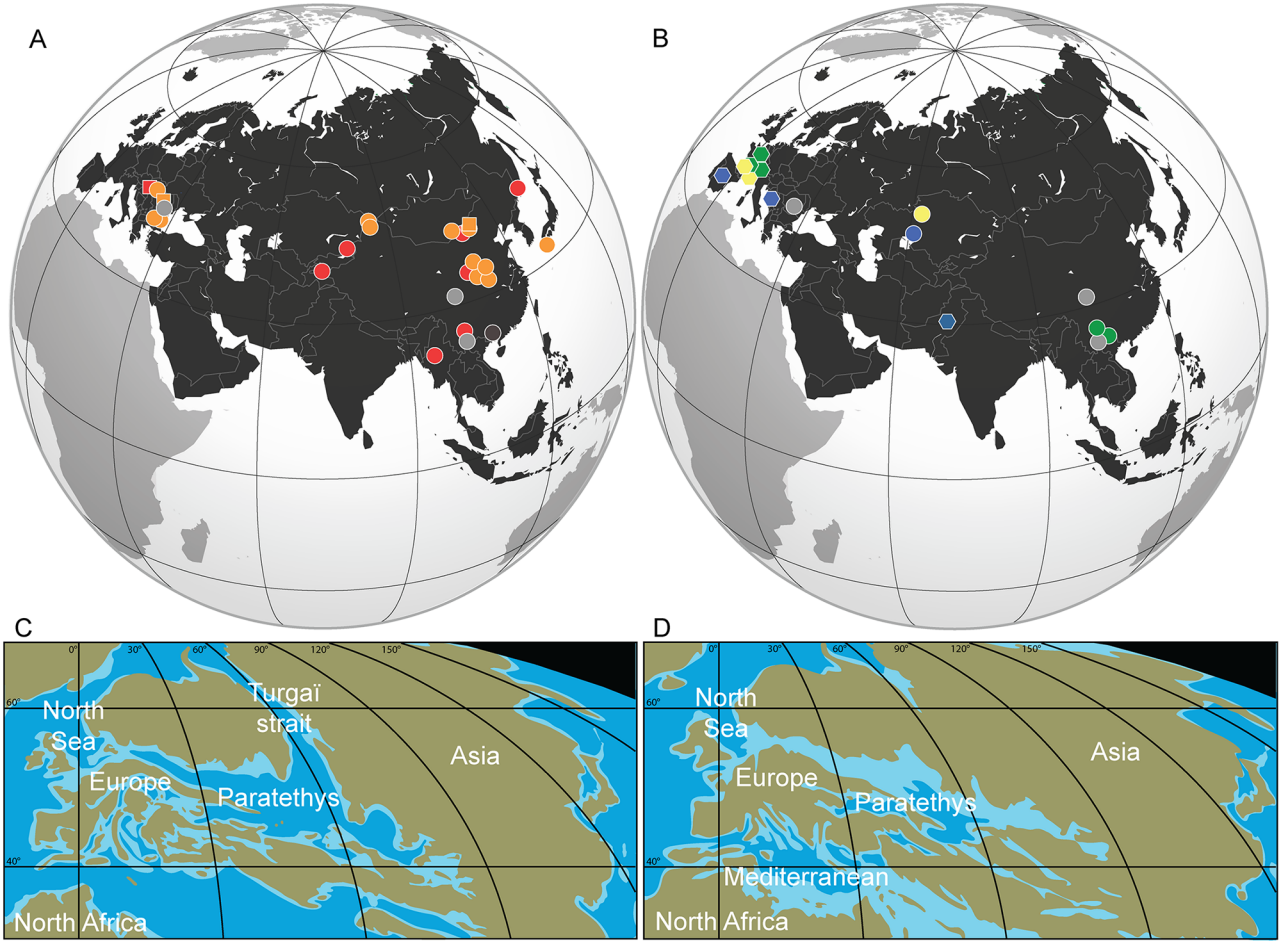
Distribution maps of Amynodontidae and palaeogeographical map reconstructions in Eurasia. Middle to Late Eocene (A) and Oligocene (B) fossil localities bearing amynodontids. See Table 4 for symbols and colours explanations as well as localities and occurrences references. Palaeogeographical map reconstructions during the Late Eocene (C) and Early Oligocene (D) modified from Ron Blakey (jan.ucc.nau.edu/rcb7/mollglobe.html).

Based on these new data and according to the distribution maps of Amynodontidae from the late Middle Eocene to Oligocene in Eurasia (Fig 9, Table 4), their presence is evidenced in South Alpine-Carpathian Europe before any other representative of the family is known in Western Europe.

**Table 4:** List of occurrences of *Cadurcotherium*, *Amynodontopsis* and other genera of Amynodontidae in Eurasia, range from Middle Eocene to Late Oligocene.

Ages		Taxa	Countries	Localities	References
Oligocene (Fig 9B)	Late Oligocene	*Cadurcotherium* (blue hexagons)	Spain	Carrascosa del Campo	Crusafont Pairó & Aguirre, 1973 [123]
			Bosnia and Herzegovina	Ugljevik	Malez & Thenius, 1985 [124]
			Pakistan	Bugti Hills	Antoine et al., 2004 [9]
		Other (blue circle)	Kazakhstan	Akespe	Bayshashov, 1994 [128]; Lucas & Emry, 1996 [54]
	MP25	*Cadurcotherium* (yellow hexagons)	France	Aubenas-les-Alpes	Ménouret et al., 2015 [129]
				Garouillas	Bonis, 1995 [15]
		Other (yellow circle)	Kazakhstan	Myneske-Suyek	Birjukov, 1961 [56]; Lucas & Emry, 1996 [54]
	Early Oligocene	*Cadurcotherium* (green hexagons)	France	Etampes	Ginsburg & Hugueney, 1987 [130]
				Isle-sur-Sorgues	Roman & Joleaud, 1909 [14]
				Phosphorites du Quercy	Gervais, 1873 [13]; Roman & Joleaud, 1909 [14]
				Moissac	Roman & Joleaud, 1909 [14]
			Switzerland	Bressaucourt	Becker, 2009 [16]
		Other (green circles)	China	Lunan Basin	Chow et al., 1964 [131]
				Qujing	Xu, 1961 [132]
?Late Eocene—?Early Oligocene (Fig 9A and 9B)		Other (grey circles)	Romania	Dobârca	This study.
			China	Lantian	Xu, 1965 [55]
				Maguan	Qi, 1992 [133]
Eocene (Fig 9A)	Late Eocene	*Amynodontopsis* (orange squares)	Romania	Morlaca	This study
			China	Urtyn Obo, Ulan Gochu	Wall, 1989 [2]; Wang, 1992 [127]
		Other (orange circles)	Bulgaria	Nikolaevo	Nikolov & Heissig, 1985 [11]
				Kameno	
			Hungary	Tápiószele	Kretzoi, 1940 [12]
			Japan	Karatsu	Tomida & Yamakasi, 1996 [134]
			Kazakhstan	Kiin Kerish	Belyaeva, 1971; Lucas et al., 1996 [89]; Emry et al., 1998 [135]
				Kamalkpay Mountain	Lucas & Emry, 1996 [54]; Emry et al., 1998 [135]
			China	Yuanqu	Young, 1937 [82]; Chow & Xu, 1965 [136]
				Lushi	Chow & Xu, 1965[136]
				Jiyuan	
				Mianchi	
				Erlian Basin	Xu, 1966 [5]; Lucas et al., 1996 [89]
			Mongolia	Ergil Obo	Gromova, 1954 [53]
	Middle-Late Eocene	Other (black circle)	China	Maoming	Averianov et al., 2016 [4]
	Middle Eocene	*Amynodontopsis* (red square)	Hungary	Dorog	This study
		Other (red circles)	Russia	Artyom	Gromova, 1960 [137]
			Myanmar	Myaing	Colbert, 1938 [79]
			Kyrgyzstan	Andarak	Averianov & Godinot, 2005 [138]
			Kazakhstan	Kyzyl Murun	Lucas & Emry, 2001 [83]
			China	Baise	Ding et al., 1977
				Erlian Basin	Osborn, 1936 [81]; Wall & Manning, 1986 [19]; Wall, 1989 [2]
				Rencun	Huang & Wang, 2001 [7]
				Weinan	Li, 2003 [8]

Occurrences with corresponding symbols and colours are also reported on Fig 9.

The palaeogeographic setting explains this situation, because from the late Middle Eocene to the earliest Oligocene (i.e. to MP20) Western Europe was isolated from Northern Europe by the North Sea and Northern Europe from Asia by the Turgaï Strait [16,18,112,139–141]. Regarding South Alpine-Carpathian Europe, it was mostly disconnected from the rest of Europe by the Perialpine and Paratethys seas—they may have been in connection at some point [142,143]—but connected to Asia [112,140,141]. Perissodactyl faunas in this part of Europe seem more similar to those found in Asia (e.g. the reported amynodontid *Amynodontopsis*, as well as *Amynodon hungaricus* [12] and *Cadurcodon ardynensis* [11], but also Brontotheridae such as *Brachydiastematherium transylvanicum* in Hungary [144] or *Sivatitanops* in Bulgaria [11], and “Hyracodontidae” like *Prohyracodon orientale* [31,145] than to those found in North and Western Europe [11]. Excluding a few sporadic and enigmatic reports [11,142], typical late Eocene Western European taxa such as *Palaeotherium* or *Plagiolophus* are indeed absent from South Alpine-Carpathian Europe. The extension phase of the distribution of Amynodontidae to Western Europe occurred only after the Eocene-Oligocene transition (Fig 9B). However, this new distribution is strictly limited to the genus *Cadurcotherium*, unknown before the Oligocene. The occurrence of this genus in Western Europe, along with *Epiaceratherium*, *Eggysodon* or *Ronzotherium* (e.g. [16,38,116,146]), represents the typical record of the post-Grande Coupure fauna (*sensu* [17]). The dispersal routes used by these new immigrants in Western Europe through North Alpine (due to the drying up of the North and Turgaï Seas) and/or South Alpine dispersal routes (due to the closure of the Paratethys and Perialpine seas)—is still debated [16,142,147]. Considering that none of the typically Eastern-European Eocene perissodactyls has ever been found in Western Europe, even after the Grande Coupure, they should not be regarded as “precursors” of the post-Grande Coupure faunas, but rather as representatives of a palaeobiogeographical extension in Eastern Europe of North Americano-Asiatic faunas during the Late Eocene. These observations suggest that, at least for rhinocerotoids, an arrival at the very beginning of the Oligocene through the northern route is more likely, but should be quantitatively investigated. A similar case is evidenced for the family Anthracotheriidae (Artiodactyla) whose early dispersal in the Balkan area and the Italian peninsula, during the Late Eocene, is recorded by taxa that differ from the newly arrived taxa in Western Europe after the Grande Coupure [148,149], as well as for ruminants, for which a South-Alpine route is suggested during the “Bachitherium Event” [150].

## Supporting information

S1 File Morphological data matrix used for the phylogenetic analysis in NEXUS format and TNT format. (ZIP)

S2 File List of synapomorphies retrieved during the phylogenetic analysis with PAUP, in Excel format. Changes in blue with double-arrows are unambiguous. Number of steps and CI of characters are also included. Numbers on the tree are node numbers. Refer to the included tree for node numbers and to the morphological data matrix for characters description (S1 File).
(XLSX)
